# Contemporary Views of the Extraction, Health Benefits, and Industrial Integration of Rice Bran Oil: A Prominent Ingredient for Holistic Human Health

**DOI:** 10.3390/foods13091305

**Published:** 2024-04-24

**Authors:** Tabussam Tufail, Huma Bader Ul Ain, Jin Chen, Muhammad Safiullah Virk, Zahoor Ahmed, Jawad Ashraf, Noor Ul Ain Shahid, Bin Xu

**Affiliations:** 1 School of Food, Biological Engineering Jiangsu University, Zhenjiang 212013, China; tabussam.tufail@dnsc.uol.edu.pk (T.T.); jchen0304@163.com (J.C.); safiullahvirk@hotmail.com (M.S.V.); zahoorhn444@gmail.com (Z.A.); jawadashraf1@outlook.com (J.A.); 2 University Institute of Diet and Nutritional Sciences, The University of Lahore, Lahore 54590, Pakistan; huma.badar@dnsc.uol.edu.pk (H.B.U.A.); aneeshahid1@gmail.com (N.U.A.S.)

**Keywords:** rice by-product, cereal characterization, bioactive components, food industry, production and health benefits

## Abstract

Globally, 50% of people consume rice (*Oryza sativa*), which is among the most abundant and extensively ingested cereal grains. Rice bran is a by-product of the cereal industry and is also considered a beneficial waste product of the rice processing industry. Rice bran oil (RBO) is created from rice bran (20–25 wt% in rice bran), which is the outermost layer of the rice kernel; has a lipid content of up to 25%; and is a considerable source of a plethora of bioactive components. The main components of RBO include high levels of fiber and phytochemicals, including vitamins, oryzanols, fatty acids, and phenolic compounds, which are beneficial to human health and well-being. This article summarizes the stabilization and extraction processes of rice bran oil from rice bran using different techniques (including solvent extraction, microwaving, ohmic heating, supercritical fluid extraction, and ultrasonication). Some studies have elaborated the various biological activities linked with RBO, such as antioxidant, anti-platelet, analgesic, anti-inflammatory, anti-thrombotic, anti-mutagenic, aphrodisiac, anti-depressant, anti-emetic, fibrinolytic, and cytotoxic activities. Due to the broad spectrum of biological activities and economic benefits of RBO, the current review article focuses on the extraction process of RBO, its bioactive components, and the potential health benefits of RBO. Furthermore, the limitations of existing studies are highlighted, and suggestions are provided for future applications of RBO as a functional food ingredient.

## 1. Introduction

Rice is Asia’s most significant cereal crop, and most people in this region consume it. Asia, America, and Africa are the primary rice-producing continents. Paddy milling yields 70% rice (endosperm) as the primary product, along with 20% rice husk, 8% rice fiber, and 2% rice germ [1,2]. Dietary elements in rice, such as carbohydrates, proteins, and fats, are vital ingredients in people’s daily meals, and rice is one of the world’s primary food supplies for half of the world’s people. It is used as a staple food in many countries, including Bangladesh. Endogenous lipids are a tiny though significant category of rice macronutrients, providing greater functional and physiological benefits than carbohydrates and proteins [3].

The brownish part of rice is known as rice bran, which is separated into refined grains during dehusking paddy grains. Rice bran is the aleurone- and pericarp-composed rigid outer layer of rice, which contains an assortment of essential nutrients such as oryzanols, tocotrienols, tocopherols, and phytosterols. Compositional analysis has shown that it contains 20–25% oil, 12–15% protein, 40–50% carbohydrates (predominantly glucose), and dietary fibers like glucan, pectin, and gum [4,5].

Rice bran (RB), once primarily utilized for animal nutrition, is now used to extract rice bran oil (RBO) [6]. Rice bran has a lipid content of up to 25%, often referred to as rice bran oil (RBO), and is a native source of various bioactive substances [7]. The predominant nations for producing oil from RB are India and Thailand. The solvent extraction of 40 million tonnes of RB produces approximately 6.5 million tonnes of RBO in India [8]. The production of RBO is predicted to be over 722 thousand metric tons among the top three manufacturers: India, China, and Japan. Small rice mills process more than half of the rice produced, providing 20 to 25 million metric tons (MMTs) of bran for the extraction of RBO, with a 3 to 4 MMT capacity for RBO. Moreover, the RBO refining industry produces gum sludge, wax sludge, and soap stock as residues, which are good sources of numerous functional and nutraceutical components, including oryzanols, tocotrienols, tocopherols, inositol, ferulic acid, lecithin, and wax [6]. Different rice varieties have different proportions of RB; for example, colored rice is rich in bioactive compounds, such as antioxidants, polyphenols, carotenoids, and tocopherol, which prevent oxidative damage to body tissue and DNA [9].

**Figure 1:**
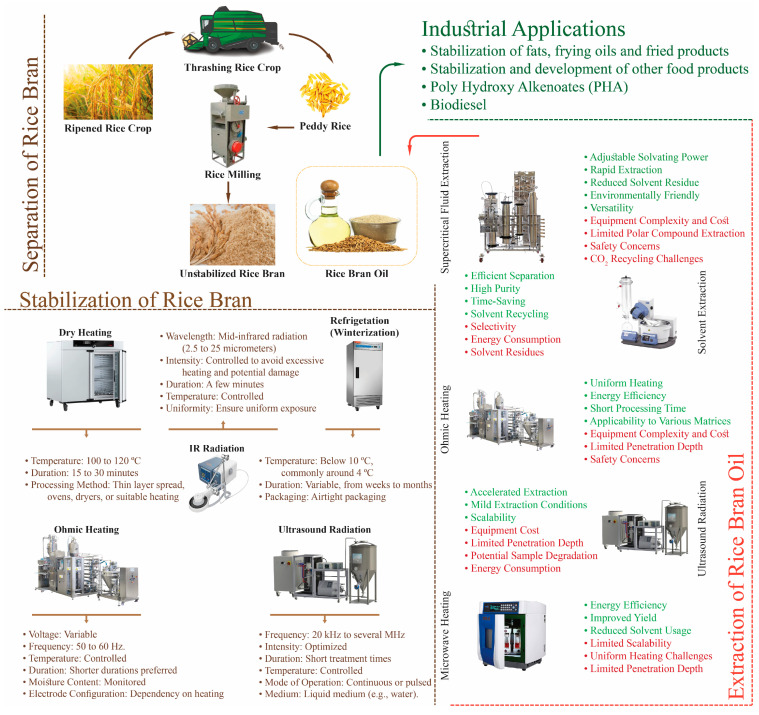
Schematic diagram of all steps followed from rice crop to refine rice bran oil.

Unsaturated fats (FAs), primarily found in RB, embryos, and endosperm portions, make up most of the fats in foods and maintain rice grain’s storage, manufacturing, and cooking properties. RBO is rich in numerous phytonutrients with established nutritional value, including γ-oryzanol, tocotrienols, and tocopherols. It also has balanced unsaturated (ω-3 and linolenic acid) and saturated (palmitic) FA combinations. RBO is a unique source of γ-oryzanol, distinct from many other industrial vegetable oils, and has gained widespread recognition for its superior nutritional efficiency [7]. RBO is also, therefore, thought to be a beneficial edible oil, having substantial nutritional content and serving as a crucial component of many therapeutic and industrial goods [10]. An overview of the whole article is represented in Figure 1 as a schematic diagram.

The prime objective of this article is to explain the methods of stabilization, extraction, and purification of oil and to explore the nutritional and health-providing aspects of RBO. Rice bran is a waste product of the cereal industry and a bioactive substance of nutritional importance and industrial applications.

## 2. Stabilization of Rice Bran

Rice bran has been an underutilized waste product of the cereal industry and is considered a by-product primarily valuable for animal feed. It has been observed to be a source of nutrition and valuable products due to the presence of functional components and endogenous lipase enzymes produced by microbial activity, which is activated during milling [11]. These lipases hydrolyze the RBO into glycerol and free fatty acids, imparting a rancid odor and acrid flavor, rendering it unfit for human consumption. RB degrades into an unpalatable substance during conventional milling in approximately six hours, rendering it unfit for humans. Due to the rancidity, most RB is used as a high-protein animal feed, fertilizer, or fuel [12]. Therefore, it is necessary to preserve RB, since oxidative changes negatively impact the RBO’s quality and are slow to manifest. These efforts destroy or inhibit lipases and reduce the loss of RBO, hence balancing the free fatty acid (FFA) content [13]. Rice bran can be preserved through several techniques, including frigid storage, sun-drying, steaming, expelling, and chemical stabilizers, such as sodium metabisulphate. In gas-permeable packaging, extrusion-stabilized RB can be securely stored at 22 °C for up to one year. However, the safest storage period for par-boiled RB is estimated to be less than three to four months [11].

Ohmic heating is another promising technique for stabilizing RB. In this technique, the sample’s electrical resistance, the passage of alternating current through a food sample, causes ohmic or electrical heating [14]. Ohmic heat stabilization of RB and increasing RBO yield, as much as 92%, is effective for bran stabilization when coupled with moisture addition. Moreover, the delayed increase in FFA concentration without a corresponding temperature rise, indicative of the nonthermal effect of electricity on lipase activity, is another advantage of ohmic heating [15].

Recently, an excellent innovation is using acids with antioxidative properties, such as ascorbic, ascorbyl palmitate, and phosphoric acid, as well as their mixtures, with food products containing parboiled RB (0.1–2.0% weight basis) to uphold the product’s stability for at least six months at room temperature. Other instances include rosmarinic acid and acacetin as well as phenolics, including cinnamic and trans-cinnamic, chlorogenic, salicylic, synaptic, quinic, ferulic, gallic, p-coumaric, vanillic acid, and caffeic acids. It has been discovered that an antioxidant composition such as “Petox” (a combination of BHA, BHT, and citric acid) is ineffective when solely used [14,16].

### 2.1. Stabilization Techniques of Rice Bran Oil

Rice bran is an abundant source of RBO, but it is susceptible to oxidation and needs to be processed as soon as possible after rice refining. Different stabilization methods and their advantages and disadvantages are given in Table 1.

#### 2.1.1. Microwave Heating

Microwaves are advantageous because they produce internal combustion of molecules at the energy-penetrating level, which renders them ideal for the thermal processing of RB with poor heat conductivity. In contrast to oven drying and steam retorting, microwave radiation promotes the dipole spin of water’s free molecules, disrupting fragile hydrogen bonds. The dipole force generates molecular friction, which warms the bran evenly across the microwave’s penetrating depths. Regarding FFA, microwave-stabilized RB performs as well as steam-retorted bran. The moisture of RB is critical in microwave stabilization. Raising the microwave energy to a particular limit aids in the stabilization of bran, which has an elevated moisture content [25].

A previous study demonstrates that the acid reading for microwave-heated RB over an 8-week storage period at 25 °C did not significantly increase compared with raw RB. The expense of microwave-heating systems has also decreased dramatically due to technological breakthroughs in this field. Furthermore, according to physical, thermal, and nutritional investigations, microwave heating inactivates lipase, preventing RB deterioration and increasing FFA. At present, the available literature about the influence of microwave heating on the color, odor, and physicochemical characteristics of the RBO, as well as its impact on micronutrients, is extensive [25].

#### 2.1.2. Extrusion

Extrusion has become one of the earliest and most widely used methods of RB stabilization, in which temperatures from 120 and 130 °C are employed to deactivate lipase. Furthermore, post-extrusion time for holding does not influence FFA concentrations in extruded RB. In a study conducted for one year, the FFA content of crude RB (70%) remained much higher than treated RB, which was below seven percent at all extrusion temperatures [26].

Extrusion is an effective technique for stabilizing RB. Over a year of preservation, the FFA content of extruded RB (140 °C) remains 20 times lower than that of raw bran. Increasing the extrusion temperatures from 110 °C to 140 °C improves stabilization. It is commonly employed in RB stabilization because of its ease of use and efficiency. Another perk of extrusion is the structural harm to RB while processing, resulting in a better RBO extraction [27].

#### 2.1.3. Dry Heating

Dry heating is a successful method for stabilization, and the final product can be stored in the refrigerator for a maximum of sixteen weeks with no anti-nutritional impacts or additional quality complications. According to the available information, dry heating is the most effective approach for stabilizing RB. Dry-heat-stabilized RB preserves its FFA content below 10% during the 8-week storage. The bran heated for one hour at 100 °C gives rise to 40% FFA contents during four weeks of storage. As the existing data are limited, additional studies are required to determine the most efficient stabilization settings to ensure that the RB’s FFA content remains under 5% after a 4-week storage time [28].

Hot air is employed to lower the amount of moisture in RB, resulting in lipase deactivation and RB stabilization. Prolonged storage and water re-absorption within RB will lessen the inhibitory impact, resulting in resumed lipase action on fat and the breakdown of numerous FFA. Therefore, moisture and temperature management are vital parameters to provide the ideal conditions for drying RB at 100–130 °C. In large-scale industrialized dry heating operations, uniform heating, heating time frame, and energy usage should be considered. Hot air drying is an excellent approach for stabilizing RB, but the procedure is expensive and unsuitable for small-scale applications. Basic heat treatment methods such as pan roasting and microwave heating are often utilized to inhibit lipase activity and extend the expected lifespan of RB [29].

#### 2.1.4. Infrared Heating

Infrared heating is another excellent method for stabilizing RB, which could be stabilized using infrared radiation at 125 V to 135 V, lasting for 5–10 min. The stabilization by IR heating at 125 V (5.4 A) lasting 10 min or 135 V (5.8 A) lasting 5 min decreases the amount of moisture, lipase action, and FFA while retaining the pre-storage amount of FFA for the whole 8-week duration of storage at 38 °C. In the meantime, the concentrations of γ-oryzanol and alpha-tocopherol minimally reduce after preservation; however, the color of RB with RBO deteriorates. During preservation, the RBO color brightens through IR therapy at 135 V over 5 min, reaching the color of unstabilized RB. As a result, IR heating using 135 V in 5 min seems to be the most effective therapy since it utilizes less time to stabilize RB. Moreover, γ-oryzanol, as well as the alpha-tocopherol amount in RBO, do not vary among IR-stabilized or unstabilized RB during any point during preservation; the color of RBO remains comparable to that of the control; while RBO possesses FFA of under five percent, demonstrating decreased propensity for rancidity accumulation after longer than one month of preservation [8].

A laboratory-scale ceramics IR drying apparatus could stabilize RB by raising the temperature to 85 degrees Celsius; however, selecting this temperature level remains equivocal. IR-treated RB’s lipase action remains lower than the naturally treated RB. However, the lipase action of the IR-treated bran rises dramatically during preservation for 20 days at 20 °C. Despite a changing tendency, IR-treated RB’s lipoxygenase and peroxidase activity considerably increases throughout storage. Furthermore, the FFA of the IR-treated materials grows considerably during storage, albeit at a slower rate than control samples [30].

#### 2.1.5. Low-Temperature Treatment

Low-temperature procedures, such as refrigeration, are also effective methods for stabilizing RB [31], owing to inhibiting lipase activity at low temperatures. Lipase function does not decrease significantly at 4 °C and 18 °C after 72 h of therapy. However, after 72 h of chilling at 80 °C, the corresponding activity reduces to about 59%. Lipase function probably decreases due to denaturation of proteins at ultra-low temperatures. Therefore, low-temperature therapy is appropriate for small and medium storage but not for prolonged storage since shorter storage is energy-efficient and cost-effective [31].

#### 2.1.6. Ohmic Heating

Ohmic heating involves passing an alternate electrical current throughout a material that conducts electricity via electrodes, raising its temperature via Joules. Food with electric conductivities ranging from 0.1 to 10 S/m are deemed appropriate for ohmic heating utilizing fields of electricity less than 100 V per cm. Because the electrodes are in close touch with the food, inert substances are essential to avoid contamination of the goods [32]. Whereas 50 or 60 Hz electrical field frequency is commonly employed because it is readily available via electricity lines, larger frequencies up to 1 megahertz can minimize unwanted responses at the food–electrode contact point [32]. In ohmic heating, the motion of ions serves as the cause for heating, but in microwaves, dipole motion, mainly from water molecules, plays a role in temperature growth [33].

The percentage of moisture in RB during ohmic heating is an essential element that determines the effectiveness of the stabilization procedure. Ohmic heating is a potential method for stabilizing RBO; nevertheless, additional studies are required to unveil its effectiveness and dynamics during prolonged storage [28]. Ohmic heating is an efficient approach for RB stabilization. In a study conducted for 75 days of preservation, FFA content spiked in ohmically heated materials compared to the unprocessed RB sample. However, after 75 days, the peroxide concentration and acidic concentrations in the ohmic heated sample remained at 4.7 meq/kg and 9.34%, respectively [14].

#### 2.1.7. Biological Treatment

Enzyme therapy, another name for biological treatment, preserves the vital nutrients and nutraceuticals found in RB, allowing it to be an ingredient in functional foods. Enzyme therapy has also demonstrated a considerable boost in prebiotic function via producing SCFAs, such as acetates and propionates [13]. The inhibition of lipase, along with lipoxygenase activity, assists in preserving the decreased form of ubiquinol-10 within the enzyme-treated RB. Although RB is still not widely utilized as a meal or healthy dietary supplement, there is significant potential for employing processed RB as a supplementary ingredient in food formulations to improve the nutritional content and health advantages [23].

#### 2.1.8. Moisture Heating

When hot steam is added to RB, its temperature rises, and lipase deactivates, rendering stability to the RB. The key benefits of moist heating over dry heating are homogeneous heating, brief heating duration, and effective inactivation of enzymes. Superheated steam for processing RB leads to the entire deactivation of lipase, and the RB can be kept for 24 weeks at 21 °C or for 12 weeks at 38 °C comprising a small amount of FFA, demonstrating that the moisture therapy technique deactivates lipase enzyme and extend the shelf life of RB [34].

Table 2 represents different stabilization techniques. Stabilizing RB could be achieved in numerous other ways involving steam therapy, which has an appropriate influence on stabilizing RBO. When contrasted with unstabilized RB, steam processing emerges as an effective means of stabilization, efficiently limiting the lipolytic action of RB and reducing FFA levels. Furthermore, steam therapy reduces oil extraction by just 3% compared to 89% in unstabilized RB. Compared with unstabilized RB, stabilization by roasting, steaming, hot air, and microwave heating effectively delays forming an acidic atmosphere, compelling controlled FFA and peroxide contents [29].

## 3. Extraction of Rice Bran Oil

Extraction utilizing non-polar liquids, like hexane, has evolved into the favored industrial procedure for commercial purposes. However, the Japanese initially extracted RBO from RB using the hydraulic press method, which still prevails [41].

**Figure 2:**
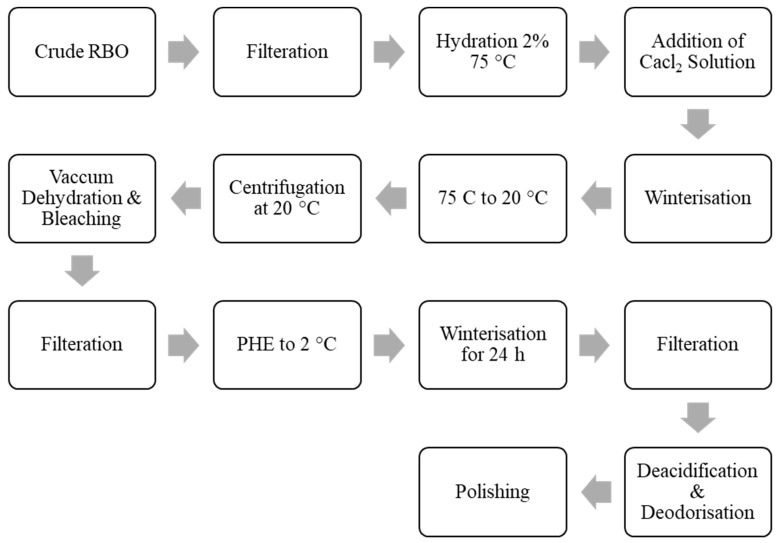
Physical refining process of rice bran oil.

The flow diagram of the physical refining of RBO is shown in Figure 2. Several procedures have been used to extract RBO from RB, such as supercritical carbon dioxide fluid extraction, compressed liquefied petroleum gas, ultrasound-assisted aqueous extraction [42], and sub-critical water extraction techniques [40]. For the commercial extraction of oil from RB, hexane is the solvent of choice [43]. Vacuum evaporation of solvent from the miscella yields crude RBO. However, hexane poses several drawbacks, including potential fire, health, and environmental hazards. Short-chain alcohols such as isopropanol and ethanol have been proposed as alternatives owing to their excellent safety and lower need for regulation [44]. Therefore, alcohol-extracted oils have more significant amounts of phosphatide and unsaponifiable compounds [1]. Ethanol has been used to extract RBO-containing E and B vitamins, while isopropanol has been used to extract RBO primarily rich in B vitamins [9].

**Figure 3:**
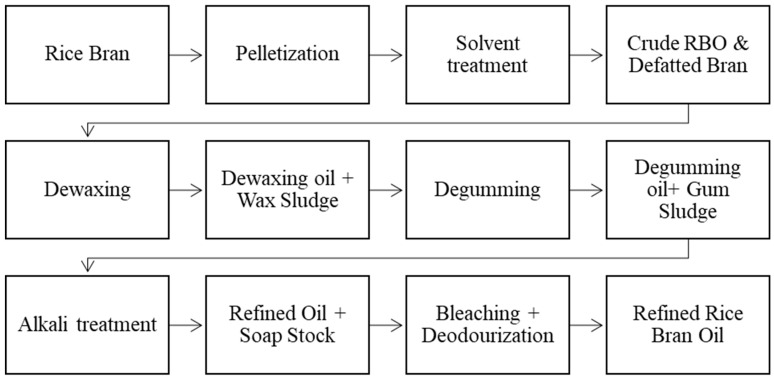
Process for the chemical refining of rice bran oil.

Though widely accepted, extraction with hexane achieves limited success regarding sound color quality by limiting FFA content because specific group components of oil seed lipids cannot be controlled. Supercritical fluid has more versatile solvent properties than liquid extraction agents [45]. This can be attributed to greater control over lipid solubility and mass transfer properties, such as the diffusivity of the extraction medium. The regulation of these properties is expanded to the entire domain of pressure and temperature above the critical point of SCF being used [46]. RBO yield with SC-CO_2_ ranges between 19.2% and 20.4%, and the yield increases with temperature at isobaric conditions [47]. Despite obvious advantages, this technology has limitations due to the high equipment cost for extraction [48]. Introducing one or two enzymatic reactions before solvent extraction results in higher oil yields. However, when these enzymatic treatments are used alone, the process does not significantly increase oil yields [41]. Cellulase and pectinase extraction with n-hexane show excellent effects of enzyme concentration for determining oil and protein extraction yields; however, incubation time and temperature show a little significant effect [49]. Figure 3 explains that Chemical refining is an alternative enzymatic reaction that uses alpha-amylase to gelatinize starch before a saccharifying step. At the same time, the residual paste containing 66.75% of the original RB may be subjected to a proteolytic process to extract proteins or directly treated with solvents to obtain RBO [50].

After refining, the RBO from the RB is stable and fit for consumption. The capacity of the present continuous-type RBO mill is 50–200 t/d, and that of the batch-type mill is 30 t/d. Refining RBO improves the quality of edible oil, is economical, and produces byproducts like oryzanol, inositol, and phytosterols, which are of pharmaceutical importance. It minimizes the problems of liquid waste and conserves energy [41,45].

### 3.1. Mechanical Pressing (Cold Pressing)

In the past, manual compression has been the most widely used technique for obtaining oil from oilseeds worldwide. In certain nations, it has been used with the help of medium and small-sized oil separation enterprises for industrial separation of RBO. Compared to solvent extraction techniques, the approach is less costly and labor-intensive [51]. Owing to customer demand for organic and safe products, this approach, which does not include heat or chemical treatment, represents an intriguing alternative to standard procedures. Moreover, it was discovered that only 9 to 10% of the RB weight is removed by pressing for RBO harvesting using this approach [52].

#### 3.1.1. Soxhlet Extraction

Vegetable and seed oils are often extracted using the Soxhlet-based solvent extraction method. The oil is generally extracted from the oil seeds by crushing them and placing them on a fixed bed that is then subjected to a straight solution. Utilizing a solvent (heptane or petroleum ether) at 40 to 60 °C produced yields of 15–20% RBO by mass of RB [44]. Typically, hexane could be the best solvent for achieving a more accurate and maximum yield. Production of around 67.73% RBO from a Soxhlet extraction using hexane could be achieved. Conversely, in terms of γ-oryzanol and α-tocopherol, SC-CO_2_ possesses the most incredible output. There is insufficient research to support soxhlet extraction for more extensive RBO separation [52].

#### 3.1.2. Supercritical Fluid Extraction

A supercritical fluid is created when a particular fluid is exposed to both temperature and pressure over its crucial moment. In these circumstances, the fluid displays characteristics that fall between a vapor and a liquid. Supercritical fluids, on the other hand, have an approximate diffusivity and a gas-like viscosity [53]. The oil output may be improved by raising the temperature to 70 °C, the pressure to 500 bar, and the separation period. Using SC-CO_2_ extraction at 45 °C at a pressure of 35 MPa, about 22% of oil per kilogram of RB is produced. However, due to the expensive nature of the equipment, this method carries several drawbacks [52].

**Figure 4:**
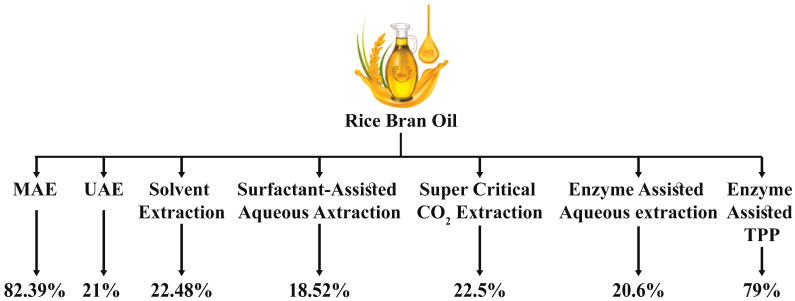
Extraction methods of rice bran oil and their total extraction yield percentage of total oil present in the rice bran.

Supercritical fluid extraction (SFE) has several advantages, notably minimal danger of solvent contamination and the ability to circumvent some of the limits of traditional extraction. SFE is more rapid and effective than organic solvent extraction due to its better penetration strength and excellent transport qualities into the matrix of materials. It relates to a separation method that employs supercritical fluid, CO_2_, at pressures and temperatures beyond the critical point, making it an appealing solvent for temperature-sensitive compounds. CO_2_ is combined with polar compounds to boost extraction efficiency and promote solubility and extraction rate [54]. SC-CO_2_ and ultrasound can both be used to increase the kinetics of RBO separation. The SC-CO_2_ extraction process produces 7.00–9.50% RBO. The SC-CO_2_ + ultrasonic extract technique is used to identify oryzanol precursors (campesterol, beta-sitosterol, stigma sterol, and 4-methylene cycloartanol) within RBO; therefore, it is suggested that this combo is a potential strategy for obtaining of selected functional chemicals [55]. Table 2 and Figure 4 represent the various methods used to extract RBO and their corresponding yield.

#### 3.1.3. Sub-Critical Fluids Extraction

“Hot liquid solvent” or “pressurized/accelerated liquid solvents” refer to sub-critical fluids employed beyond their boiling points by applying pressure while being pressed below their crucial temperatures. The solvent is eliminated following extraction using a vacuum and a low temperature in the ongoing counter-current method known as sub-critical fluid extraction. In this process, heated water is used to keep its solvent condition at a temperature level between 100 °C and its crucial index of 374 °C under a suitable pressure of around 22 MPa. This process produces oil with higher yield, excellent value, and density of healthy ingredients. Still, it is not yet commonly used, and there is no information about it being used to extract RBO [52].

#### 3.1.4. Assisting Enzymes Aqueous Extraction

The enzyme-assisted liquid separation for RBO has been considered an environmentally beneficial method of producing high-quality oil. The liberation of the oil and the protein into the aqueous system is facilitated by the enzymatic reaction, which aids in hydrolyzing and degrading the structural polysaccharides that make up the cell walls of the oils and proteins [56]. Combining enzymatic extraction with ultrasonic processing makes the production of over 92% RBO attainable. At 550 °C, 4.5 pH level, and 5.5 h hydrolysis time, enzyme hydrolysis utilizing catalysts (cellulose 1.2%, protease 0.6%, and amylase 0.3%) produces excellent results. Since it yields oil with high-quality characteristics such as color, free fatty acids, peroxide value, and phosphorus concentrations, this approach is superior to conventional procedures [52].

#### 3.1.5. Microwave-Assisted Aqueous Extraction

Microwave-assisted extraction (MAE) is recognized as a potential extraction approach for oils and other biologically active compounds because of its quick temperature increase, minimal energy and liquid consumption, and excellent yield outputs [57]. Concerning microwaves, it should be noted that electromagnetic waves are composed of perpendicularly rotating magnetic and electric fields. Heat is produced when molecular dipoles inside an item rotate under the effect of an elevated frequency electromagnetic space, like microwaves. When chemicals are separated from plants, the heat elevates the pressure, forcing the plant cell walls to break, resulting in mass transfer among the solvents used and extracted substances [36].

In a comparative study of conventional and MAE of RBO regarding productivity and product composition, utilizing MAE increases oil production by as much as 20%. If MAE is employed as a treatment, isopropanol generates the maximum oil output and oryzanol content. MAE boosts extraction efficiency concerning both oil output and key bioactive components [58]. Polar solvents, such as isopropanol and ethanol, are expected to be more successful in MAE due to their ability to respond to microwaves [36].

The microwave-assisted extraction technique has some drawbacks and advantages. It requires specific properties of solvents for each component that could sometimes be difficult, and it requires optimization of some parameters specifically for complex materials to obtain the required results. The power range for MAE is 100 to 700 watts. Typically, it depends upon the nature and scale of the unit and sample. High power may risk increasing the degradation of some sensitive compounds, and it may increase the extraction efficiency. It has been mentioned that the optimal time duration is 8–10 min that could be effective in achieving rice bran oil yields of approximately 95%, which is much better than the traditional methods like the Soxhlet extraction method, which generally takes hours to extract the oil content from rice bran.

At an extraction duration of 8 to 10 min and a solvent-to-bran proportion of 1.6–2.3 mL per g, over 95 percent of the higher quality crude oil could be obtained, compared to standard solvent extraction. Generally, MAE is recognized as a versatile and successful approach for RBO extraction, with short extraction periods, high yields, high-quality products, and good selectivity. The brief time required to acquire heat through MAE is thought to deteriorate the hydrolytic enzyme instead of increasing enzyme stimulation, preserving the recovered enzyme’s purity [59].

#### 3.1.6. Ultrasound-Assisted Extraction

Ultrasound-assisted extraction (UAE) has been frequently used. Ultrasound is conveyed as a wave of pressure across a material and induces excitation through accelerated molecular mobility. Moreover, ultrasonic treatment causes cavitation, which enhances the permeation of the tissues of plants. Microfractures and cellular wall disintegration provide additional evidence for the mechanical properties of ultrasound, allowing for the discharge of the material within [60]. UAE offers several benefits, including excellent extraction output, repeatability, minimal solvent consumption, fast extraction time, cheap running expenses, low environmental impact, and adaptability to industrial-scale applications. UAE is primarily used in food processing, such as drying, extraction, and emulsification—sonication-aided RBO extraction results in FFA and peroxide levels lower than the industry standard and increased phenolic substances. The response surface approach optimizes the UAE variables to maximize RBO production [53]. The oil obtained by UAE has less FFA and less color-conferring compounds than that obtained by hexane. Both extraction procedures provide a more significant proportion of oil using parboiled RB than raw RB [61]. Ultrasonication is a technique in which ultrasonic waves with different frequencies are employed, and resultantly, the sample collapse and cell structure disruption occur, facilitating the oil extraction. Solvent selection is a crucial step in this technique. For rice bran, n-hexane could be the effective solvent for the extraction of the highest yield of oil content, but there are some health claims associated with it. Alternatively, ethanol is safer and more effective for achieving maximum extraction yield. The optimum time of oil extraction through ultrasonication is around 30 to 45 min, and it usually depends upon the operational conditions like ultrasonication temperature, frequency, power, and solvent-to-feed ratio [60].

#### 3.1.7. Solvent Extraction

Solvent extraction is another manufacturing approach to extract nutritious oils or other chemicals. It has previously been used in a variety of industries, including the treatment of waste, food technology, pharmaceuticals, and hydrometallurgy. This extraction technique uses chemicals as solvents to remove liquid content from a solid–liquid mixture [44]. The solvent might be either nonpolar or polar. Chloroform, methanol, ethanol, hexane, acetone, petroleum ether, and dietary ether are common solvents employed in business. The ongoing flow of the countercurrent technique is the most prevalent extraction method employed in the food-grade oil extraction business. The sample flows in a particular direction, while its solvent flows in the reverse direction. The opposing current flow arrangement might increase the contact area between the sample and the solvent used. It can better use the solvents and enhance the quantity of retrieved material in the final stage. After the extraction procedure, the cake, a defatted meal, is gathered on one side. In contrast, the chemical solvent and oil-containing solution are gathered on the other side. As it recovers a high proportion of oil, the solvent extraction technique is widely utilized across the globe. In manufacturing, the solvent extraction method may reach a high yield of up to 99% recovery [56]. There are many benefits to the solvent extraction method, including quicker time for extraction with a high percentage of oil recovery, procedure simplicity, and expense efficiency [59].

Hexane provides the most effective solubilizing characteristics and a lower boiling point than other liquids for RBO separation [52]. The comparison by replacing ethanol with hexane and assessing the effect of humidity and temperature on RBO production shows that the production of RBO decreases as water content increases. Still, the production of other lipid molecules increases as temperature increases [62]. The RBO production using hexane with isopropanol at a 2:1 and 3:1 weight ratio, 60 °C or 40 °C extraction temperatures, and varied extraction times reveals that at 60 °C for 10 min, isopropanol produces slightly less oil (201.2 g per kg) compared to hexane (211 g per kg) [19].

#### 3.1.8. Surfactant-Assisted Aqueous Extraction

The surfactant-assisted aqueous extraction procedure (SAAEP) is a more ecologically friendly oil extraction technique. The optimal settings for RBO extraction from RB are determined using the reactive surface methodology (RSM) approach and the Box–Behnken design (BBD), which has three components and stages. Surfactant and oil proportions (SOR) (3, 3.5, and 4) are utilized as distinct parameters, as are ultra-sonication amplitude (60%, 70%, and 80%), and ultra-sonication contact duration (15, 20, and 25 min). In the meantime, the oil percentage is employed as the dependent variable. Furthermore, the rice bran of stabilized rice, stabilized at 4.25 percent moisture, produces over 20% recovered RBO and about 2.0 meqO_2_/kg FFA concentrations. In addition, experimental extraction at the best level used to evaluate the predicted findings shows that the extracted oil has a purity of 18.5%, which differed by around 0.81% from the projected values. Therefore, the RSM approach is practical in optimizing the RBO level [63].

#### 3.1.9. Enzyme Assisted Three Phase Partitioning

Three-phase partitioning (TTP) is a new technology that partitions hydrophobic, hydrophilic, and protein constituents into three phases: t-butanol (top stage), protein (middle stage), and ammonium sulfate (bottom stage). This approach was used initially to separate enzymes, proteins, and lipids but is currently being used to extract biological agents from plant-based sources, including oils, oleoresins, and polysaccharides. Many variables influence TPP, including salting out, isotonic precipitation, co-solvent kosmotropic, osmolytic and precipitation impact, protein hydration changes, and electrostatic attraction. TPP has various benefits over the traditional protein extraction process, including gentle operating temperatures and scalability. The procedure is also cost-effective due to low-cost ingredients, including t-butanol and ammonium sulfate. Upon processing the plant materials with a specific enzyme or a mixture of enzymes, the standard procedure is followed by enzyme-aided TPP. TPP has been used to obtain oil from three distinct substrates: mango kernel, soybean, and RB. The prior treatment with an industrial enzyme preparation, followed by TPP, corresponds to oil yields of about 98, 86, and 79 percent (*w*/*w*) for soybean, RB, and mango kernel. Since enzyme-aided extraction saves time, it is preferred over solvent extraction. Furthermore, using t-butanol as the solvent is significantly safer than using n-hexane, commonly used to extract oil [39].

## 4. Refining of Rice Bran Oil

Rice bran oil is coarsely obtained from RB and then purified using various physical and chemical procedures. This method involves several significant phases, such as filtering, degumming, dewaxing, deacidification, bleaching, and deodorization [64]. Before consumption, RBO is refined to eliminate unwanted contaminants that affect the oil’s purity and shelf-life. A large amount of NaOH is required in chemical procedures to eliminate all FFA from deodorized RBO.

Degumming, neutralizing FFA, bleaching, dewaxing, and steam deodorization are all phases in the refinement of RBO. Since it is challenging to obtain refined RBO due to the substantial amounts of FFA, waxes, unsaponifiable components, and polar lipids present in crude RBO, many refining stages are adjusted to produce high-quality RBO [65]. Crude RBO is composed of 4% unsaponifiable, 2–4% free fatty acids, and 88–89% neutral lipids. The unsaponifiable fraction is a complex mixture of naturally occurring antioxidant compounds such as vitamin E and oryzanol [41]. A flow diagram represents the process of physical refining of RBO, as shown in Figure 2.

Solvent extraction optimization and nanofiltration procedures are used to refine RBO. Because RB contains a lot of phosphorus and wax, it must first be degummed and dewaxed before refining. With low phosphorus levels, the phospholipase-A1-mediated degumming displayed a higher extraction effectiveness. Chemically refined RBO has a lesser fatty acid content than physiologically processed RBO, indicating that the refining process has a noticeable impact on the structure of the oils. Though alkali treatment eliminates 93 to 94.5% of γ-oryzanol from the original crude oil, degumming and dewaxing of crude RBO only eliminate 1.1 and 5.9%, respectively. The bioactive substances and fatty acid makeup of crude and refined RBO also vary [47].

RBO loses more γ-oryzanol and tocopherols during subsequent refinements than unsaponifiable materials [66]. The solidified soap showed the highest levels of γ-oryzanol, 14.2 mg/g, while the deodorization distillate demonstrated the highest levels of tocopherol, 5.76 mg/g, during RBO refining [67]. The structure and concentration of fatty acids, oryzanol, tocopherols, tocotrienols, and sterols impact the storage stability of RBO, which is crucial for both health and economic reasons. According to studies focusing on fatty acid composition, crude RBO has increased storage stability over polished RBO, while γ-oryzanol and sterols have higher storage stability than other RBO constituents [66].

### 4.1. Winterization

Combined with wax, RBO includes enough glycerides, which have high melting points, and saturated fats, so winterization is necessary to complete a 5 h freezing test. Oil from dewaxed rice is commonly hazy and viscous. At room temperature, it may be less without winterization. Filtration comes after cooling the oil at predetermined rates and temperatures during winterization [68]. To winterize RBO, it must first be cooled from 30 to 35 °C at a constant pace to 15 °C for twelve hours with gentle agitation. Further, it cooled to 4 to 5 °C without any agitation and held for 24 to 48 h to allow higher melting components to crystallize. The pace of chilling and temperature differences affect the crystals that form [67].

For filterability, large, stable crystals are preferred. Filter aids may help extract the particles from the thick oil. The maximum time for cold testing of winterized oil is 5–7 h. The high melting solids are more successfully separated from RBO by miscella winterization [68]. Among the solvents, isopropyl, hexane, and acetone are employed. The mixture is kept for 24–48 h before filtering after being gradually chilled over 12 h to 15 °C with stirring, then 4 °C to 5 °C without agitation. For applications involving the extraction of solids from liquids, solutes from solvents, and liquids from liquids, membrane-based separation and purification technologies have lately proven themselves effective, economical, and ecologically friendly techniques. Membrane filtration is primarily a pressure-driven, size-exclusion-based technique. Membrane composition, temperature, pressure, flow rate, and interactions among feedstock elements and the membrane surface all impact how well membrane filtration works [69].

### 4.2. Bleaching, Hydrogenation, and Deodorization

Rice bran oil is bleached, hydrogenated, and deodorized using conventional techniques. Activated carbon or bleaching soil is used for bleaching. Due to its high cost and processing challenges, activated carbon is rarely employed. The amount of bleach clay to use varies from 2% to 10% depending upon the qualities of the oil separated from RB and bleaching earth [70]. More recent silica-bleaching earths are more efficient for producing suitable oil colors. It is possible to eliminate offensive scents caused by peroxides, aldehydes, and ketones, as well as the distinctive tastes and aromas of RBO, by deodorization or steam stripping. So, the oil is kept at 220–250 °C while vacuuming to 3–5 mmHg. The most popular kinds of deodorizers are semicontinuous ones [70]. RBO that has been deodorized should be stored the same as other oils. Deacidification and deodorization are combined during physical refining, also known as steam refining. Physical refining is more effective for high-free-fatty oils and produces more neutralized oil than alkali processing [71].

### 4.3. Oxidative Stability

The oxidative stability index (OSI) of crude RBO is over 37 h, more than four times longer than that of soybean oil. It is common knowledge that γ-oryzanol is responsible for the remarkable resilience of RBO to oxidation. Items made from RBO that retain γ-oryzanol should have more excellent oxidation resistance. Apart from the raw RBO, it is difficult to observe the OSI of the RBO mixes precisely because of their stiff nature, which prevents air from evenly bubbling through the sampling. Instead, the oxidative stabilities of the combinations were assessed after the RBO spreads were dissolved at 1, 2, and 3 weight percent in purified soybean oil. Soybean oil has an OSI of 8.44 h on its own. The OSI of all RBO spreads was prolonged to about 12 h at 1% and to around 13 h at 2%. Consequently, based on the concentration, the coatings made from RBO might extend the induction time by up to 50% [72].

### 4.4. Electrostatic Filtration

**Figure 5:**
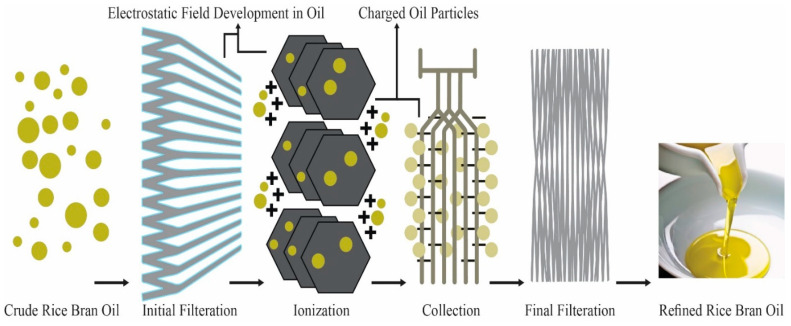
Working of electrostatic filters under a uniform electric field.

Oil pollution is always harmful to the ecosystem. Pollution is inevitable in hydraulic and lubricating system oils, which causes tribological difficulties. Several oil cleaners have been created to reduce such issues, significantly enhancing the system’s dependability. The electrostatic filters for oil are intended for oil filtering and the development of non-detergent oil and ultra-clean hydraulic oil, both of which have several uses in the industrial sector, such as compression pump oil systems. The primary function of an electrostatic filter for oil is to clean the fine suspended particulates in oil, which may reach the oil cleaner via the bottom inlet. We transmit the suspended particulates employing the electrostatic field produced by implementing high voltage across the two electrodes. The nature of the charge attracts particles that are charged to the electrodes. As oil flows laminarly from the bottom to the head of the cleansing cell, electrically charged particles gather on the electrical media, which includes separator or collector paper, and then become entangled and extracted from the oil. Electrostatic oil cleansers work because the electrodes catch impurities that float in oil with an electric charge. Positive, negative, and neutral particles float in oils and are electrically categorized. As the polluted fuel travels via high-voltage electricity, the embedded dirt contaminants are charged and directed toward the metal electrodes. A field of electricity of 14,000 V is applied among the two metal electrodes. As the operation starts, suspended particulates entrap within the collector, and every one of the particles is recovered, even those of submicronic size [73]. The whole process of electrostatic filtration is shown in Figure 5 as a schematic diagram.

## 5. Composition of Rice Bran Oil

Rice bran oil is acquired from the germ and bran of rice. Due to its high oil yield (12% to 18%) and realistic refining yield (50% to 70%), RBO is considered a valuable oil [74]. RBO is a transparent, subtle pale yellow colored and odorless oil with a subtle nutty taste. The ICMR, the WHO, the Chinese Cereals and Oils Association (CCOA), the American Heart Association (AHA), and the National Institute of Nutrition, India (NIN) consider RBO “healthy,” which makes RBO an excellent cooking oil. RBO primarily consists of unsaponifiable and saponifiable fractions, which offer various nutritional advantages. The outermost layer of a grain of rice, known as RB, weighs just 8–10% of the entire grain but comprises most of the micronutrients, including minerals (7–10%), dietary fiber (7–11%), proteins (11–15%), and fats (15–20%) [75].

RBO typically consists of diacylglycerols (2–3%), monoacylglycerols (1–2%), triglycerides (81–84%), wax (3–4%), FFAs (2–6%), phospholipids (1–2%), glycolipids (0.8%), unsaponifiable (4%), and 4hydroxy-3-methoxy cinnamic acid (FA) [64,76]. The main components of rice include carbs, proteins, and trace amounts of minerals, fiber, and moisture. Both bran and germ primarily retain minerals and vitamins [75]. Trans fats are absent from RBO. As opposed to other vegetable oils, RBO includes enough α-linolenic acid to support the de novo technique for the production of additional polyunsaturated omega-3 fats, which are healthy fats involving docosahexaenoic acid and eicosapentaenoic acid present in the tissues phospholipid bilayer. Therefore, depending on the extraction technique, the relative number of fatty acids in RBO may change (cold or hot extraction). The fatty acid composition in oils derived from vegetables varies and reacts individually while heating because of high quantities of unsaturated fatty acids and other phytochemical constituents. As a result of its usual fatty acid makeup, lesser cohesiveness, and exceeded exhaust temperature (254 °C), RBO may be used among various cooking methods. It is beneficial in salad dressings and for sautéing, barbecuing, and sauces.

Moreover, it is regarded as the ideal oil for cooking in stir frying and deep frying since it expedites cooking and can reduce energy consumption. When frying, food prepared at extreme temperatures seems to absorb less RBO by about 15% [77]. Foods prepared with RBO have superior flavor and are less greasy when consumed. Its diverse bioactive constituents and vitamin E content are antioxidants, preventing oxidation and rancidity and contributing to better thermal stability, increasing storage consistency [78]. The accessibility of α-tocopherol in deep frying may be protected by the existence of α-oryzanol and α-tocotrienol [79,80]. Different compositional analyses have been represented in Table 3.

### 5.1. Fatty Acids

Compared to the American Heart Association’s (AHA) and WHO standards, RBO has well-in-range saturated fatty acids (22%), monounsaturated fatty acids (43%), and percent polyunsaturated fatty acids (35%). RBO is considered a highly nutritious cooking oil since it has a well-balanced combination of polyunsaturated, monounsaturated, and saturated fatty acids, with a ratio of 1:1.1:0.6 [81]. The primary constituents of triglycerides in RBO consist of three fatty acids: oleic acid-C18:1, linoleic acid-C18:2, and palmitic acid-C16:0, which account for about 90% of the total fatty acid content in RBO. Oleic acid is the most prevalent fatty acid in RBO, accounting for 42% of total triglycerides, followed by linoleic acid and palmitic acid, at 32% and 20%, respectively. Palmitic acid predominates as RBO’s most common saturated fatty acid, while stearic and myristic acids are in relatively minor quantities [82].

A significant bioactive component of RBO known as γ-oryzanol is composed of cycloartenol, 24-methylene cycloartenol, -sitosterol, and campesteral 4-hydroxy-3-methoxy cinnamic acid esters. Unrefined RBO contains about 15 g/kg γ-oryzanol. The primary policosanols (PCs), fatty acid alcohols of basic structure [CH_3_(CH_2_) nCH_2_OH), as determined by gas chromatography-tandem mass spectrometry, were tetracosanol, triacontanol, hexacosanol, and octacosanol, which accounted for 87.3% (172.2 mg/kg oil) of the total PCs [81]. Phospholipids (PLs) are a small family of lipids in tiny amounts in rice grains but are essential components of cell membranes, the exterior of oleosomes, and other organelle membranes. PLs are prevalent in the embryos, bran, aleurone layer, and endosperm, accounting for up to 10% of all grain lipids. Glycerophospholipids, distinct PLs found in rice, consist of fatty acids polymerized to a phosphate molecule, a glycerol core, and a polar terminal [70].

To alter the fatty acid composition and define a formalized lipid, capric acid is lipase-catalyzed into RBO, which also contains oleic and linoleic acids, both long linear chain fatty acids with poor absorption efficiency than medium linear chain fatty acids, including capric acid, in people with fat digestion malformations [83]. In the process of lipase-catalyzed interesterification, a mixture of RBO and palm stearin produces a shortening free of trans fats and contains bioactive components, which meets the demand for hydrogenation-free vegetable oil-based shortening, which helps prevent the formation of harmful trans fatty acids. The resulting shortening from palm stearin and RBO is nutritionally superior and can be produced commercially.

### 5.2. γ-Oryzanol in Rice Bran Oil

γ-oryzanol is a group of chemicals found in RBO, wheat bran, and some vegetables and fruits. γ-oryzanol is a valuable byproduct derived from RB processing, and alkali refining impacts the secondary constituents in crude RBO, such as γ-oryzanol, FFA, RB wax, and long-chain fatty alcohols [52]. Oryzanol benefits humans, spurring interest in developing simple techniques for separating it directly from RBO soap stock produced during the RBO clarification chemical process. It is essential to examine the issues that arise during oryzanol extraction and how to resolve them, such as previous processing, compositional variations in soap stock, susceptibility to bulk transfer, water level, and surface-friendly substances that can cause emulsification. In addition, it is essential to meticulously design the engineering inputs required to address issues, such as mass transfer area improvement, saponification, and drying techniques. Utilizing TLC-densitometric and TLC-image analysis to quantify γ-oryzanol in cold-pressed RBO is advantageous [84]. The byproduct of the RBO operation, leftover from the distillation of saturated fats in oil from RB soap stock, contains a significant amount of oryzanol. Gamma-oryzanol has poor absorption in the gastrointestinal tract, showing its potential to lower cholesterol levels by decreasing the absorption of dietary cholesterol [85].

### 5.3. Vitamin E in Rice Bran Oil

Compared to other oil seeds, RBO contains more tocopherols. The unsaponifiable fraction of RBO contains approximately 1% (*v*/*v*)-tocopherol. According to an HPLC analysis of RBO, there are 3.02 mg of alpha-tocopherol per 1 g of RBO. Tocotrienols are prevalent in vegetable oils, such as RBO and palm oil [86]. The tocotrienol-rich portion, which is found in rice, barley, palm, and oat brans, contains approximately 70% tocotrienols. Dimethyl tocotrienol (d-P25-T3) and desmethyl tocotrienol (d-P21-T3) are identified and extracted from stabilized RB. Based on the origin of the bran and the industrial refining procedure, RBO is rich in tocotrienols, containing 72–1157 ppm [87]. Tocotrienol comprises about 1.7% (*v*/*v*) of the unsaponifiable portion of RBO. Moreover, 0.5 mg-tocotrienol is present in 1 g of RBO. Tocotrienols have been reported to be safe at even much higher concentrations, and consumption of 240 mg daily for two years shows no damaging consequences. Tocotrienols have increased biological activity and greater concentration than tocopherols [87]. There are four main kinds of vitamin E (tocotrienols and tocopherols) in RBO, which are, respectively, α-, β-, γ-, and δ-oryzanol. RBO contains significant amounts of tocotrienols (0.025–0.17%) and tocopherols (0.02–0.08%), which vary based on rice variety, cultivation location, and extraction technique [86,87]. Tocotrienol possesses triple-double bonds in the hydrocarbon tail at the 11′, 7′, and 3′ positions. The fluidity of tocotrienols is improved due to these double bonds, causing their effortless weaving to the cell membrane and making them more similar to highly polyunsaturated fats in edible oils. Among tocotrienols, β-tocotrienol, γ-tocotrienol (5,7,8, trimethyltocotrienols) (8-methyltocotrienol), and γ-tocotrienol (7,8-dimethyltocotrienol), are predominant [88].

### 5.4. Protein

Defatted RB is undoubtedly a possible leftover, including RB flour following the expulsion of oil from RB, which emerged due to the rising requirement for RB with its essential product, RBO, as beneficial nutritional and therapeutic ingredients. By iso-electric precipitation and alkali separation from defatted RB, RB protein (RBP) is formed, which has a true digestibility of 94.8% [80].

### 5.5. Enzymes

Several active catalysts prevail in RB, such as intensive enzymatic activity, which can be witnessed in the germ and the outermost layers of the seed coat. Several such enzymes are always in place, including lipase, alpha and beta-glycosidase, lecithinase, invertase, cytochrome oxidase, catalase, flavin oxidase, deoxyribonuclease, dehydrogenase, lipoxygen, ascorbic acid oxidase, and esterase [89]. Since they impact the maintenance of integrity and storage stability of RB, peroxidase, lipoxygenase, and lipase are the most significant in commerce. To produce glycerol and an amount of FFA, lipase encourages the hydrolysis of RBO [90]. The consistency of the oil is concentrated in aleurone and sub-aleurone linings, as well as the rice embryo, the germ of rice grain. The epicuticular region is where molecules of lipases are concentrated. Similar compartmentalization happens in the germ, as 60% of the lipase is produced. The substrates and enzymatic activity are combined during grinding. External conditions impact FFA formation, and a daily production of 5–7% FFA has been recorded. For just one month of bran storage, an FFA of up to 70% can be observed [56].

Some major bioactive compounds and their health potentials are given in Table 4.

## 6. Applications of Rice Bran Oil

In addition to being a healthy vegetable oil, RBO is exceptional, with high qualities and several health advantages. It may be utilized for frying, making fat and shortening, and making advanced nutritious oils as it has good stability, delectable flavor, and lengthy fry property life [4]. More significantly, RBO has substantial potential in its application in producing medicines and cosmetics. Remarkably high concentrations of oryzanol, sitosterol, other plant sterols, fat-soluble vitamins, and other micronutrients are found in RBO [50]. As a result, research on RBO has focused on its role and use as an edible oil for a family’s daily health in various nations worldwide [2].

### 6.1. Cooking

Rice bran oil is a mild-flavored oil that may be utilized in various cuisines. It differs from rapeseed and soybean oils, which have aggressive and strong qualities that may mask the food’s original flavor. Moreover, RBO has an extremely high smoke point (350 °C) [66]. As a result, it cooks with little degradation or polymerization and is relatively stable. RBO’s viscosity is another distinctive quality. The most viscous of the different oils, RBO, contains >10,000 mg/kg oryzanol, which may impact viscosity, taking it even higher than extra virgin olive oil. RBO’s viscosity renders excellent dressing ability, particularly in various Chinese dishes. Because of its high thickness, RBO is easily held on food surfaces, giving it a glossy, delicious appearance [96].

### 6.2. Deep Frying

Because of elevated ignition point, durability, and distinct frying attributes, RBO is more frequently utilized for frying purposes than other oils, reducing the amount of oil needed [7]. As an excellent frying and salad oil with strong oxidation durability due to its increased number of different tocopherols and tocotrienols (860 ppm), purified RBO plays a significant role in food preparation. A “deep fat frying” procedure involves submerging food in a sizable amount of hot oil or fat. Regular frying times range from 5 to 10 min, and temperature varies from 175 to 195 °C [97]. Concerning the fried meal’s quality, the frying oil’s composition is crucial since it can offer certain distinctive organoleptic and sensory qualities, such as flavor, textures, and sight [2]. RBO is the recommended oil for frying and baking due to its strong oxidative stability. Snacks prepared in RBO absorb 12–25% less oil than those made with ground nut oil, making RBO ideal for regular cooking and deep frying. A further benefit of RBO is that food cooks more quickly, uses less oil, and still has excellent oxidative stability and holding qualities [79].

### 6.3. Flavor

RBO produces a nice, sweet flavor attributed to vanillin’s probable existence when heated. Among the most popular flavoring ingredients, vanillin is produced after the breakdown of RBO-oryzanol into ferulic acid and sterols/triterpene alcohols, especially after heating [80]. Vanillin’s predecessor is ferulic acid, which comes from oryzanol, giving heated RBO its pleasant flavor. However, the precise chemical pathway or mechanism has yet to be described. In contrast to other vegetable oils, RBO creates less of a foul flavor component (acrolein) that gives food a disagreeable flavor when cooked. RBO costs three times as much as other edible oils in Japan since it emits a pleasant scent when heated. However, RBO has continued to be a widely applied frying oil in several Japanese restaurants [52].

### 6.4. Functional Foods Applications

As a functional ingredient, RB is a source of oryzanol, ferulic acid, tocotrienol, tocopherol, and adenosine. Recently, with the increasing attention to RBO, its practical characteristics, and its therapeutic properties, scientists/consumers can devise ways to add it to their diets as a functional food. These characteristics may inspire businesspeople to manufacture RBO-based meals or different products on a considerable scale and satisfy the world’s desire for superfoods and functional foods [98]. Dyslipidemia has been successfully treated with RB and its metabolites in several animals. Because treated RB contains known active ingredients such as oryzanols, tocotrienols, tocopherols, nucleotides, phenolic compounds, and dietary fiber, an assortment of health-promoting products have been produced from it. By reducing inflammation, dyslipidemia, and hypertension and acting as a powerful functional dietary ingredient to minimize cell damage, biotechnological therapies like the enzymatic hydrolysis of RB are beneficial against dyslipidemia [99].

Several other powerful advantageous effects of RB are due to an excellent source of phytonutrients. These phytonutrients may modify lifestyle-related illness by diminishing digestive disorders and hypertension, possibly by restoring balanced blood pressure and oxidative stress, suppressing cell growth, and decreasing inflammatory response, according to evidence from prior in vivo and in vitro studies [99]. Due to its extraordinary nutritional and therapeutic value, RB improves overall health, lowers the risk of illness, and enhances life quality. So, RB may be regarded as a healthy or valuable food. As a result, there is a high need for beneficial bran ingredient enrichment in various diet-based treatments that decrease inflammation and illnesses associated with a sedentary lifestyle. Creating effective RB dietary treatments and evaluating their efficacy in lowering the presence of lifestyle-related illness and inflammation-associated biomarkers should thus be the goal of future scientific research [98].

### 6.5. Health Benefits of Rice Bran Oil

Generally, RBO contains significantly high oryzanol, fat-soluble vitamins, sitosterol, various phytonutrients, and other nutrients, among other nutritional elements [100]. Furthermore, RBO has been employed as a component in health-promoting active meals and has several health-endorsing benefits in cells for both people and animals that may be related to unsaponifiable substances, notably oryzanol [55]. Punia et al. also explain the detailed research on the health benefits of RBO, as shown in Table 5.

**Figure 6:**
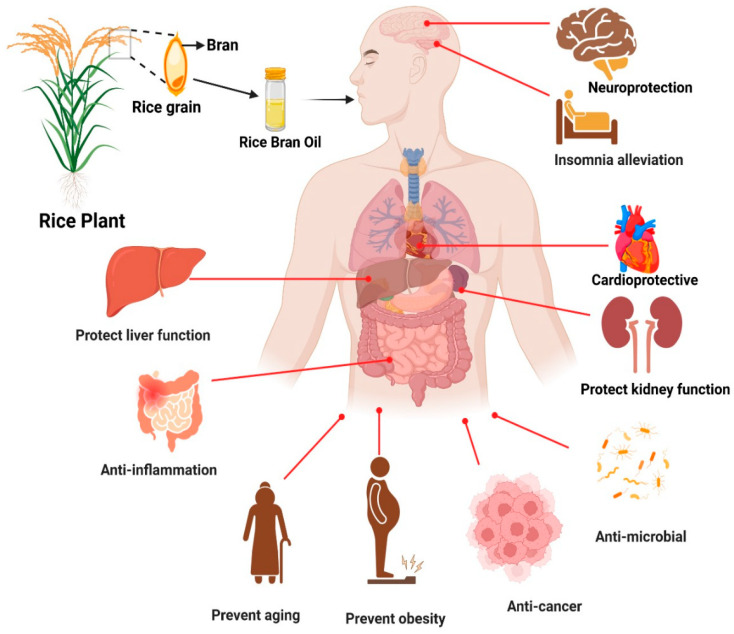
Health-endorsing perspectives of rice bran oil.

Moreover, RBO also increases cholesterol metabolism and decreases cholesterol absorption in the gut, suggesting that it may prevent lipid irregularities and lower the risk of heart disease. RBO can directly or indirectly decrease caspase activity, preventing beta-cell death and lowering the chance of developing hyperglycemia. Figure 6 represents the overview of the health benefits of RBO [100].

In comparison to certain other commonly utilized oils for cooking, including safflower, sunflower, canola, corn, and olive oils, RBO is becoming more famous due to its exceptional cooking properties, extended storage life, maintained lower fatty acid development, and the existence of several biologically active components [96]. RBO is recognized as “premium edible oil” in several Asian cultures, where it is widely utilized. In Japan, RBO is frequently called “oil for the heart” but is increasingly recognized as a “healthy food” in Western nations. It is also gaining demand in the USA and is popular in other countries owing to its affordable cost and numerous health advantages [100].

#### 6.5.1. Antihyperlipidemic

Hyperlipidemia is characterized by aberrant lipid metabolism in the human body, defined by elevated levels of one or several plasma lipids produced by a disruption in the expression of specific genes. In previous studies, including clinical trials, garlic extracts, glycosides, flavonoids, and other polysaccharides have been scientifically proven to reduce plasma lipid levels effectively [105]. Supplementation of RBO can mitigate the hyperlipidemic symptoms induced by HFD in mice, including enhancements in serum lipid levels, body weight, adipose tissue size, and liver weight. The RBO supplement also improves the extent of fatty degeneration in liver tissues and regulates the expression levels of genes associated with lipid metabolism. Readily available and cost-effective RBO offers a novel and encouraging approach to treating hyperlipidemia [102]. The mode of action of RBO on fat metabolism’s impact remains unidentified. The presence of tocotrienols, phytosterols, and triterpene alcohols γ-oryzanol in RBO at high concentrations has been discovered to have beneficial effects on fatty acid synthesis, which is attributed to their ability to scavenge harmful substances, reduce hyperlipidemia, and prevent the formation of atherosclerosis. The anti-hyperlipidemic effects of RBO can be attributed to its distinctive bioactive compounds, while other types of fatty acids (poly and monounsaturated) also seem to have an impact. The phytonutrients, specifically ss-sitosterol and 4-desmethylsterols (excluding 4,4′-dimethylsterols), have been proven to lower blood TC and LDL-C concentrations in RBO. Phytosterols can potentially affect the absorption of dietary cholesterol in the gut and the binding of cholesterol to bile acids for excretion in fecal matter [105,106].

**Figure 7:**
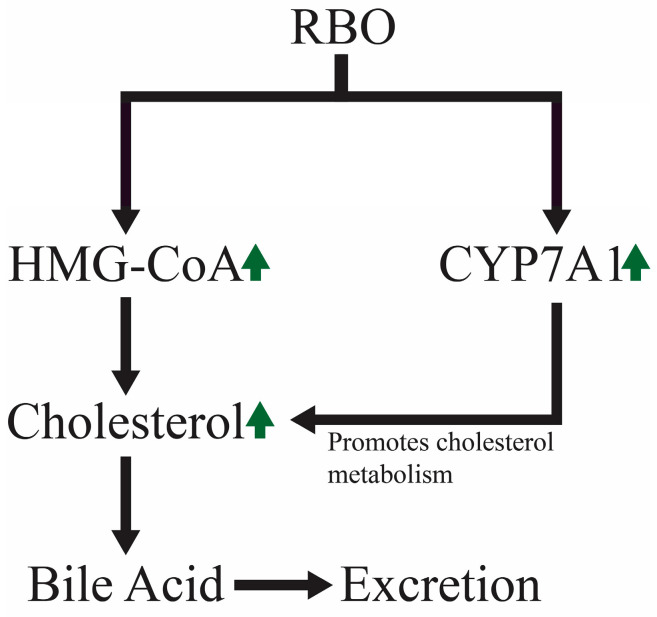
Cholesterol-lowering mechanism of rice bran oil [100].

Figure 7 represents the basic cholesterol-lowering mechanism as a schematic diagram. Non-human primates have also been used in studies of RBO’s hypolipidemic response. The blood levels of apolipoprotein-B, LDL-C, and TC significantly decreased when RBO or its mixes were used as dietary fat at 20–25% of total calories consumed. Practically, purified RBO’s (PRBO) ability to reduce cholesterol may be due to reduced cholesterol absorption rather than hepatic cholesterol production. They hypothesized that PRBO’s non-triglyceride portion may indeed be responsible for a decrease in the production of fatty streaks, one of the first indications of arteriosclerosis [107]. While comparing the ability of various vegetable oils to decrease cholesterol, researchers exhibit the palliative effects of several bioactive RBO components individually, including γ-oryzanol and trans-ferulic acid. Hamsters fed on a high-cholesterol diet (HCD), HCD plus 10% RBO, HCD plus 0.5% trans-ferulic acid, and HCD plus 0.5% γ-oryzanol show that diets containing 10 percent RBO, 0.5 percent trans ferulic acid, and 0.5 percent γ-oryzanol have significantly lower blood very low density lipoprotein+ low-density lipoprotein and total serum levels with cholesterol than the comparison groups [108]. Plasma lipid hydroperoxides and triglycerides significantly reduce in the mice feeding on meals containing γ-oryzanol and RBO. In comparison to trans-ferulic acid, the findings indicate how γ-oryzanol may improve the reduction of blood lipoprotein with very low density and low-density lipoprotein levels and may also aid in enhancing the high-density lipoprotein cholesterol levels [109]. An overview of health-endorsing perspectives of RBO is given in Figure 6.

Patients with chronic kidney disease (CKD) taking RBO show a significant improvement in high-density lipoprotein and a reduction in very-low-density lipoprotein, low-density lipoprotein, triglycerides, and overall cholesterol levels. RBO supplementation of the diet and medication management of dyslipidemia reduce death in patients with CKD [110]. Participants with hypocholesterolemia consuming cooked rice and using brown rice extract daily, with inositol, GABA, and oryzanol as primary components, exhibit considerably diminished blood lipids concentrations (including cholesterol level, beta lipoprotein, as well as low-density lipoprotein cholesterol). This trend mainly prevails in people whose baseline total cholesterol levels remain over 200 mg/dL, especially regarding bad cholesterol. [111]. By boosting the production of 7-alpha hydroxylase (CYP7A1) of cholesterol and encouraging cholesterol breakdown, it helps lower LDL cholesterol. To balance bodily cholesterol, 3-hydroxy-3-methyl glutaryl coenzyme A reductase expression increases cholesterol production. It has been established that the HMG-CoA reductase activity can be decreased by tocotrienol in RBO as low as 300–500%, compared to coconut oil [112].

#### 6.5.2. Antioxidant Property of RBO

There are several issues with aging in the food sector. Lipid oxidation is unquestionably among the food industry’s complicated problems to solve. Even though phytochemicals like butylated hydroxytoluene (BHT) and butylated hydroxy anisole (BHA) have shown effectiveness in enhancing oxidative stability, their use is still not widely accepted because of the alleged adverse health consequences. As a result, γ-oryzanol is a fantastic choice when looking for natural antioxidants utilized in cuisines [113]. The ferulic extract in γ-oryzanol is closely linked to its antioxidant properties. Like many other phenolic antioxidants, ferulic acid is a potent radical scavenger that captures and stabilizes radical species, reducing the harmful impact of reactive oxygen species. (ROS). The bonded ferulic molecule inside γ-oryzanol is more stable at high temperatures even though ferulic acid has a more vital antioxidative ability since the larger molecular weight reduces its tendency to vaporize at high temperatures [114].

Because of its ample supply of nutritionally significant phytoceuticals, including oryzanol, tocotrienols, and tocopherols, among many others, RBO stands out from other edible oils. Oryzanol can be employed in medicines, cosmeceuticals, and nutraceuticals. It is a potent antioxidant prevalent in RBO at a ratio of 1% to 2%. Oryzanol comprises many different forms of phytosterols with ferulic esters [115]. The only justification for employing oryzanol in many domains, such as decreasing blood cholesterol levels, is its antioxidant properties. This antioxidant potential is unveiled in the latest research using three methodologies: the reducing power test, the nitric oxide scavenging test, and the DPPH shielding test. According to the findings, oryzanol has very significant antioxidant activity, making it a potential addition to human nutrition to combat several illnesses brought on by excessive cholesterol levels, including hypercholesterolemia, arteriosclerosis, and others [116]. Distinct physicochemical and antioxidant qualities could be found in the RBO sample prepared from RB of several distinct rice varieties, comprising colored and uncolored white rice varieties. Colored rice (KDML 105) has superior antioxidant properties over white rice (HN and RJM). Moreover, the extraction techniques impact outputs, physicochemical makeup, and antioxidant qualities of RBO specimens. The SC-CO_2_ removal produces the maximum RBO output, and RBO made from the HN-RB processed using SC-CO_2_ has the maximum antioxidant potential [117]. An overview of health-endorsing perspectives of RBO is given in Figure 6.

#### 6.5.3. Anti-Diabetic Effects of Rice Bran Oil

Numerous medications exist, considering the significant influence of diabetes’ rising prevalence due to a lack of therapies that are both cost-effective and efficient. However, artificial anti-diabetic medications have a wide range of adverse effects, including lactic acidosis, diarrhea, and hepatic issues. As a result, the current emphasis of scientists and researchers is on finding better accessible organic therapies with therapeutic potential and no adverse effects [118]. In addition to administering insulin, nutritional treatments, which improve healthy fiber intake and decrease ingestion of processed and refined carbohydrates, lower postprandial glycosuria. Such methods also effectively lower blood sugar levels by making cells more sensitive to insulin and slowing down digestion. These actions, however, remain unable to control the fluctuation in blood glucose levels to the necessary degree. The potential role of nutraceuticals in this context has been thoroughly investigated. The constituents of RB demonstrate a promising ability to reduce hyperglycemia and related disorders [119].

Furthermore, essential micronutrients in RB, γ-oryzanol, tocols, and dilatory fibers considerably decreased the diabetic state and its effects by controlling the function of liver enzymes that control sugar metabolism and proteins engaged in the insulin signaling pathway. Extensive studies on dosage, therapy duration, and other related nutraceutical operations are required. In both developing and industrialized nations, creating functional foods dependent on RB and raising public knowledge of the usage of products obtained from RB in everyday life may assist in controlling hyperglycemia more effectively [118].

Evidence from mouse models and human diabetes individuals’ medical trials demonstrate that the tocotrienol-rich portion of RBO can reduce blood sugar levels. Because they block the action of the enzyme HMG-CoA reductase inside the metabolic processes of lipid metabolism, tocotrienols in RBO are thought to reduce plasma TC levels. Tocotrienols can decrease heart attacks and improve the post-ischemic ventricular state, which gives them cardioprotective qualities. The active compounds and antioxidants found in RBO, together with its oleic acid and covalently linked linoleic acid (CLA) concentration, can aid in boosting the body’s metabolism, regulating sugar levels and lipid panel, lowering inflammation, losing weight, and controlling obesity [109].

Individuals with type 2 diabetes taking sesame oil with RBO as their only source of dietary fat for eight weeks leads to substantial reductions in fasting and postprandial sugar levels at weeks four and eight. In type 2 diabetes mellitus patients receiving sesame oil and RBO, Glycosylated hemoglobin, TC, triglyceride, and LDL cholesterol dramatically lower at week 8, whereas high-density lipoprotein levels significantly rise [120]. RBO consumption improves blood lipid profiles, but its precise mechanism of action on diabetic hyperlipidemia and the onset of hyperglycemia is yet unknown. Hence, more research is required to see whether it has any possible health advantages for treating and managing diabetes and elevated blood glucose levels [109].

#### 6.5.4. Antibacterial Activity of Rice Bran Oil

Purified RBO and isolated cycloartenyl ferulate show antibacterial action towards various plant pathogens in the antibacterial properties testing. Cycloartenyl ferulate may reduce H_2_O_2_-induced peroxidation. The complete usage of RB, representing a considerable loss of an agricultural product, is possible by isolating γ-oryzanol from RBO. Additionally, γ-oryzanol is potentially an effective biological fungicide and neuro-protectant [121]. The antibacterial and antioxidant properties of 14 vegetable edible oils sold in Japan exhibit suppression of the two bacteria’s growth, and the inhibitory values are proportionate to the dosages used, which indicates that such oils have antibacterial properties. Even though the inhibitory amplitudes are more significant than the negative controls in the *S. aureus* experiment at dosages of 0.01–0.05 mg/mL, no discernible variation emerges. Cotton, sesame, RB, grape, and chia have noticeably increasing levels of suppression than the adverse control at the maximal dosage of 0.10 mg/mL [55].

Many people utilize edible oils, which have tremendous pharmacological and therapeutic qualities. The use of oils with antibacterial properties against human diseases is possible. Research into the antibacterial properties, mechanism of action, and prospective applications of oils has picked up steam. In nations with low per capita income, there is a resurgence of employing conventional methods to guard against diseases, insects, and deterioration in cattle and food ingredients. Therefore, RBO, readily accessible on the market, is used to feed the bacterial spores, and the growth parameters are sustained according to standard procedure, demonstrating an antimicrobial impact for a more extended period [122].

Moreover, considering their potential negative impacts on foods, chemical stabilizers have been questioned regarding overall sustainability. Alternative supply stabilizers that have a superior safety record may be natural substances. In contrast to the chemical preservative calcium propionate, none of the phenolic chemicals from RBO exhibit cytotoxic action when tested for antimicrobial and anticytotoxic (*Penicillium verrucosum*) characteristics of biomass generated by Rhizopus Oryza as well as from *Spirulina* sp. In NIH/3T3 cells. Phenolics from cultured RB plus *Spirulina* sp. LEB-18 inhibits the development of fungi by 39.8% and 20.2%, respectively, and ochratoxin A production by 40.2% and 29%. The effectiveness of the phenolics from RB and spirulina remains substantially greater at 2.5 and 1.5, correspondingly, whenever the impacts of phenolics are matched with that of calcium propionate [123].

#### 6.5.5. Anticancer Effects of Rice Bran Oil

Different genes dysregulate in carcinoma, which causes a range of unpleasant side effects. Suitable anti-inflammatory dietary components, which inhibit NF-kB initiation and inflammation-related inflammatory processes, may reduce the signs of carcinoma as disruption of inflammatory pathways, which leads to elevated concentrations of the pro-inflammatory cytokines, like IL-6 as well as NF-kB is linked to different malignancies [124]. Fermented RB and RB lysates are potential medicinal substances for preventing and treating carcinoma. They could be essential in reducing localized inflammation, stopping the proliferation of tumor cells, encouraging tumorigenesis, and boosting anti-chemotherapy activities. The regulation of the gut’s microbiota’s diversity, maintaining intestinal function, mitigating adverse effects, and enhancing the medicinal benefits in people with cancer are additional advantages of RB within food [125].

After decreasing blood cholesterol, cancer prevention may be the possible medical advantage of RBO or its components, which is most frequently highlighted. The concentration of scavengers in RBO, also primarily attributable to tocols and oryzanol, serves as one of the main reasons it is thought to have the ability to prevent malignancy. Oral intake of tocotrienols significantly reduces pulmonary and liver cancer development in rats. In patients with hepatocellular carcinoma HepG2 cells, delta-tocotrienols demonstrate more notable anti-proliferative effects than the other tocotrienols [126].

Apoptosis, also referred to as programmable cell death, is a tightly controlled procedure for cell self-destruction. Based on the source of the cellular impulses driving the action, the external path (death receptor apoptotic path) and the intrinsic pathway (mitochondrial apoptotic pathway) may be included in a lethal signaling pathway. MUFAs found in RBO are anticancer in nature. The peroxisome proliferator-activated receptors (PPAR) may be the mechanism within which chained linoleic acid (CLA) exerts its immune-enhancing effects. PPAR-γ is found in several carcinoma cell lines, including elevated lipoma, breast, colonic, pancreatic, urinary bladder, prostatic, and gastric malignancies. CLA may increase PPAR action, and PPAR N-end phosphorylation and p65 can decrease NF-kB action, encouraging death and reducing cellular proliferation. By altering the peanut acid signals driving TNF-, CLA can block c-myc and enhance the production of p53 and caspase. Even though the method by which TNF kills tumors is unknown and the procedure is somewhat sluggish, it can be exploited as a possible tumor treatment [127].

Active elements in RBO induce apoptosis in colonic cancerous cells, breast cancer cells, and pancreatic cancerous cells. Palm oil plus linoleic acid can cause hep-2 cells to apoptosis via triggering ER stress. Moreover, RBO can induce apoptosis in linoleic acid and HepG2 cells, and the apoptosis-inducing activity of RBO in HepG2 is superior to that in linoleic acid alone, which could be due to the synergistic action of monounsaturated fatty acids or other anti-carcinogenic components in RBO [128].

In an animal model of inflammatory mediators’ carcinogenesis, regressed QR-32 cells develop into deadly tumors, fermenting brown rice plus RB with 5% or 10% Aspergillus oryzae (FBRA) significantly decrease cancer occurrences at 35% and 20%, respectively. Moreover, FBRA reduces the number of inflammatory cells penetrating the sponges; however, injection of FBRA does not result in myelosuppression, proving that the drug’s anti-inflammatory actions are localized to the inflamed region [129]. It is thought that fermented brown rice plus RB containing Aspergillus oryzae (FBRA) may also stop experimentally induced tumorigenesis in several rat organs. Male transgender rats for adeno-cancer of the prostate (TRAP) 6-week-old rodents feeding on meals comprising 5% or 10% FBRA for fifteen weeks have slow development of prostate cancer and reduced frequency of adenocarcinoma in the laterally prostate. In histopathological elevated prostatic intraepithelial neoplasia, therapy with FBRA promotes death and reduces cell growth. Within the prostate of mice feeding on a diet enriched with FBRA, phospho-AMP-activated kinase (Thr172) slightly elevates, which shows that FBRA seems to have the capacity to be translated into the treatment of human prostate cancer through controlling tumor development by triggering mechanisms sensitive to caloric restriction [130].

#### 6.5.6. Colorectal Cancer

Rice bran oil has the potential to reduce the cause of colorectal cancer, specifically when it is growing. It may be helpful if some of this oil could be added to our daily eating. The facts demonstrate that phytoestrogens can prevent chemically caused cancers, and according to some studies, a small amount of this element is available in RBO. The colonic microbiota produces coprostanol and some other benign sterols plus bile salts using serum cholesterol, which links to colonic cancer. Secondary bile salt compounds also aid colonic cancer growth. Nutritional phytoestrogens reduce the multiplication of epithelial cells, which significantly impacts the levels of fecal matter cholesterol, cholesterol decomposition intermediates, and bile acids, which could increase elimination of cholesterol itself along with a reduction of microbial fermentation of cholesterol and/or secondary bile acids in the gut [1,131].

Nutritional fiber ingestion and the prevalence of colorectal carcinogenesis were compared by Bingham et al. Inside a group of participants categorized by gender-specific, cohort-wide deciles, but from linear models connecting the risk proportion ratio to fiber ingestion represented as a continuous variable, the quantity of dietary fiber ingestion provided the adjusted hazard predictions. The findings demonstrated an antagonistic relationship between dietary fiber consumption and the development of colorectal cancer. The left side of the colon had the most prominent protective role, while the rectum displayed the lowest protective role. The corrected relative risk was 0.58 for the greatest percentile of soluble fiber compared to the lowest quintile (0.41–0.85). Thus, it was concluded that the incidence of large bowel carcinoma was significantly lowered by 40 percent when people with a low level of average dietary soluble fiber ingestion approximately doubled their total fiber consumption [132,133].

#### 6.5.7. Regulating Immune Response

Linoleic, a vital omega-6 fatty acid, is abundant in RBO. Due to a rise in eicosanoids generated from omega-6 fatty acids, it is believed that n-6 fats have pro-inflammatory implications. Additionally, γ-oryzanol could alter immune function, but it is not the cause of the overall immunostimulant action of RBO. Therefore, in circumstances where a stimulatory effect of the immunological response is necessary, RBO-enriched meals would be helpful [134].

The growth, management, and ideal body’s immune performance can be impacted by diet and dietary conditions. Because of its numerous favorable physiological impacts on human well-being, oryzanol, a principal bioactive constituent of RBO, significantly increases antibody titer values and enhances the postponed type of hypersensitive response brought on by sheep red blood cells. Additionally, it dramatically reduces myelosuppression in individuals with cyclophosphamide and increases the phagocytic indices in the carbon elimination test. The reports show how OZ can improve immunological function through cellular and humoral-mediated pathways [135].

There is mounting evidence that nutritional elements control inflammation, but relatively little is understood about the specific processes by which this happens. Through oxidation and consequent mitochondrial biogenesis, healthy fats represent a significant power source for cellular metabolism. Thus, the effects of RBO treatment on mitochondrial function, cytokine secretion, and the expression of activation markers show that in murine macrophages, RBO increases mitochondrial function while reducing inflammatory reactions. Nevertheless, RBO includes other substances besides lipids, such as tocopherols and oryzanol, among various others. For forthcoming comprehensive contributions and connections on the anti-inflammatory properties, investigative research, as well as medical examinations for confirmation of animal outcomes, are necessary [136].

#### 6.5.8. The Antihypertension Activity of Rice Bran Oil

Antihypertension is a state in which the body’s ability to neutralize free radicals produced by numerous metabolic processes is surpassed. The moderate fatty acids included in RBO have good benefits in reducing the formation of free radicals. Ultimately, such fats help decrease lipids’ degradation [101]. All these occurrences have a lipid-lowering and hypertension impact. Additionally, it stimulates the production of epithelium nitric oxide and inhibits the stimulation of the NF-kB pathway and angiotensin-converting factor [55]. A persistent, gradable, and separate vulnerability for cardiovascular disorders is hypertension (CVDs). It is widely known that high blood pressure is associated with both CVD death and morbidity [109].

Unsaturated fats, including antioxidants, sesame oil, and RBO, are well recognized for lowering cardiovascular disease risk. In people with mild-to-moderate hypertension, using a mixture of 20% cold-pressed, raw sesame oil high in lignin and 80% mechanically purified RBO for sixty days as a sole source of cooking oil decreases systolic, diastolic, and average arterial pressure significantly. Moreover, the subjects taking sesame oil and RBO mixed with nifedipine showed the most significant drop in blood pressure [137]. Furthermore, direct consumption of VRBO (virgin rice bran oil) shields from high blood pressure, oxidative stress, and inflammatory processes brought along by L-NAME-induced high blood pressure in mice. The inhibition of ACE, Nicotinamide adenine dinucleotide phosphate, and NF-kB mechanisms and the stimulation of eNOS could be responsible for VRBO’s antihypertensive effects. It is fascinating to note that VRBO and Lis therapy combined are more successful than either VRBO or Lis therapy separately, suggesting VRBO as a high blood pressure adjunct medication or even as a preventative measure [113].

Regular ingestion of 30 g of RBO as a portion of the daily recommended intake of fat can indeed positively affect LVEF (left ventricular ejection fraction) as well as other CAD-related biochemical parameters, such as non-HDL lipid, triglyceride, blood glucose, and uric acid, as well as reduce inflammatory responses in men with CAD over the course about an eight-week prosecution. Nevertheless, RBO has little appreciable impact on HDL levels or blood pressure conditions. Adopting food management within therapy techniques is necessary due to the hidden effects it has on enhancing the outcome of CAD. Therefore, replacing conventional oil in regular healthy eating with RBO, a nutritious oil, might be a feasible adjunct therapy for CAD patients receiving catheters [138].

#### 6.5.9. Antiaging Effects of Rice Bran Oil

The oryzanol constituent also protects against peroxidation caused by ultraviolet radiation and may be utilized as a powerful sunblock agent. Gamma oryzanol contains ferulic acid and its esters, which promote hair development and slow down the aging process of the epidermis. Tocotrienol levels in RB are around 500 ppm, which, when applied to the skin, quickly enter and are consumed. Because of their antioxidant properties, they build up in the stratum corneum of the epidermis and serve as the initial layer of protection. They control the reactive species that the skin produces when it is subjected to oxidizing radiation. They aid skin regeneration by shielding the skin from UV-induced aging and degradation. Tocotrienols can be added to sunblock to increase their effectiveness by reducing or absorbing UV light [1].

Tiny dispersions are useful in many business sectors, including the chemical, pharmaceutical, and cosmetic industries. The anti-aging, skin condition, and sunblock businesses all employ RBO in their compositions. The RBO microemulsion enhances the moisturizing deviation by almost 38% in community members with healthy skin and, therefore, by 30% in volunteer groups with atopic dermatitis or psoriasis, which is an acceptable outcome considering that significant commercial moisturizers only enhance skin moisture by almost 20% after fourteen days of usage. The probable reason for these improved results is the tiny dispersion particles sticking to the epidermis and generating a thick coating that prevents water evaporation. Following therapy for 30 min, the oiliness indices of the epidermis handled with micro dispersion rise significantly before declining within the regular and afflicted skin categories. The formulation’s 10% RBO content may cause a rise. In cases of dry skin, makeup dispersions provide an oily coating on the epidermis that might shield the lipid boundary. Consequently, the nano-emulsion’s oiliness may offer a different form of psoriasis therapy [139].

The anti-aging effects of the gel and cream compositions (cream nio and cream RBO) having RBO lysates with essential components F, O, and P bound in noisome demonstrate not solely the excitation of fibroblast development and suppression of mitochondrial membrane potential-2 activity by semi-purified RB harvests comprising F, O, as well as P, along with the betterment of skin characteristics such as water intake, discoloration, thickness and harshness, and skin elasticity mainly on the skin for just 28 days following the application of gel nio as well as cream nio. Cream RBO’s impressive effectiveness also supports the combination skin-antiaging benefits of RB bioactive substances confined in noisome. Furthermore, the extracts’ durability improves by encapsulating the beneficial components in niosomes and incorporating them into gel or cream compositions. The combination of niosomes, RB active ingredients, and the ingredients in gels and cream preparations may provide anti-aging skin benefits. It is recommended that innovative anti-aging compositions with improved in vivo and in vitro anti-aging efficacy be used on normal and non-itchy skin [140].

Sunlight that emits too much ultraviolet light damages the epidermis by inducing inflammation and oxidation, which might result in burns, photodamage, and malignancy. A hydrogel comprising RBO-filled liposomes nanocapsules, having an average diameter of 200 nm, a negative electrostatic capacity of 9 mV, and low polydispersity indices of 0.20, could reduce ear edema brought on by UVB irradiation by 60–90% compared to gel comprising LNC made using a combination of medium-chain lipids rather than RB. Hydrogel comprising the nano-encapsulated RBO decreases the protein carbonylation quantities (an indicator of oxidative stress) and NF-kB nuclear translocation (an indicator of pro-inflammatory and carcinogenic action) by 81% and 87%, respectively, showing how microencapsulation might enhance RBO’s protective qualities against skin irritation brought on by Uvb radiation exposure [141]. The findings indicate that fine-tuning is required to achieve commercial quality standards for durable, massive metals and bacterial contamination-free, rice phytochemical-based beauty goods [142]. Table 4 represents the health-endorsing perspectives of RBO. An overview of health-endorsing perspectives of RBO is given in Figure 6.

#### 6.5.10. Other Cosmetic Applications

For several years, rice has been touted as a hypoallergic food, so it stands to reason that RBO would likewise be hypoallergic, which justifies RBO’s widespread usage in personal care products. Furthermore, more research needs to be conducted to support RBO’s hypoallergic claims. Diet-specific proteins technically cause allergies, whereas highly processed oil does not include proteins. The sole exception is cold-pressed oils, which may contain traces of proteinaceous remnants. However, solvents are often used to remove the majority of commercially sold RBO. Undiluted RBO, plus hydrolyzed RB wax, are not allergens when testing animal skin and eyes. The Skincare Component Evaluation Expert Panel concludes that even these rice-derived compounds are healthy when used as cosmetic ingredients under the reported conditions of practices, applications, and quantities [143].

Furthermore, RBO is a moisturizer for babies, particularly those with delicate skin. RBO, on the contrary, is utilized in skin lotions and soaps that promise to reduce aging and the development of face wrinkling, since it is high in antioxidants like vitamin E and gamma-oryzanols. Japanese ladies are known as Nuka-Bijin when they use RBO on their faces to maintain their skin’s smoothness and radiance (bran beauties). Massaging lubricants on the marketplace are now made from RB, apricot, sesame, jojoba, and nut oils. RBO is also utilized in several other cosmetic products, including lipstick, sunblock, and conditioner since it prevents UV radiation from causing melanin hyperpigmentation [144]. The tocotrienol content of RBO, which is around 500 ppb, is the most important mechanism. Tocotrienols enter and quickly dissolve into the skin after administration. The skin stores those tocotrienols, which function as the body’s initial layer of protection and have antioxidant qualities. As a result, tocotrienols help to control the reactive species that are produced whenever the skin is subjected to oxidizing radiation. Tocotrienols will, therefore, offer defense against sun-induced damage to the skin [96].

#### 6.5.11. Neuroprotective Effects of Rice Bran Oil

As mentioned earlier, phytochemicals are significant bioactive components in RB and RBO. Sitosterol, cycloartenyl ferulate, 24-methylenecycloartanyl ferulate, cycloartenyl ferulate, plus campesterol ferulate found in RB and RBO express dopamine neuroprotection and the underlying factors. The dopamine neuron may be partly protected by the enhanced cellular oxidative tolerance brought on by the stimulation of both the DAF-16/FOXO network and suppression of the apoptosis protein CED-3 upregulation, which provides renewed information on the bioactive components of various phytonutrients beneficial for further nutritious analysis of RB and RBO [145].

A triacyl-glyceride known as 1,3-dipalmitoyl-2-oleoylglycerol (POP), discovered in lubricants derived from a variety of natural origins, such as RB, sunflower seeds, as well as palm kernels, taken directly at doses of 1, 3, and 5 mg/kg three times—0.5 h prior to middle cerebral artery occlusion (MCAO), 1 h post-MCAO, as well as 1 h after reperfusion—markedly decreases the infarct/edema volume and neurobehavioral impairments caused by the middle cerebral artery occlusion/reperfusion (MCAO/R) paradigm in mice. Moreover, POP treatment stops the oxidation breakdown of triglycerides and glutathione reduction that MCAO/R brings into the mice’s brains. POP treatment also prevents the dysregulation of phosphorylation protein kinase B, phosphorylation cyclic (adenosine monophosphate) AMP response element-binding protein (CREB), as well as phosphatidylinositol 3′-kinase in the ischemic brain. POP may also show neuroprotective benefits by blocking p38 MAPK and activating the PI3K/Akt/CREB network, which has antioxidants, antiapoptotic, and anti-inflammatory properties [146]. In older-age people, Alzheimer’s disease (AD) is indeed the most common reason for dementia. The main characteristics of AD that cause neurological dysfunction include baseline frontal brain cholinergic nerve losses and intracellular neurofibrillary tangles, including hyperphosphorylation proteins (p-tau) and external cellular neurotic plaques made of β-amyloid peptides (A) [147].

The Dokkham, Dokkha, and variants, which already have significant bioactive component levels and antioxidant capacities, have also been studied for their neuroprotective and neurogenic properties. Dokkha, discovered to have substantial neuroprotective properties, could result from substances apart from γ-oryzanol in the Dokkha isolate and might even be employed as a novel natural product of nutritionally rich material. In conjunction with oryzanol, Khemngen and Dokkha also contain additives that can increase neurite quantity and development. All the isolates and γ-oryzanol demonstrate neurogenic properties by encouraging rises in the number of nerve fibers, suggesting the possibility of using RBO as a nutritional supplement [148].

One of the most severe adverse consequences of long-term anti-psychiatric therapy is tardive dyskinesia (TD). Vapid chewing motions are aberrant mouth motions brought on by long-term neuroleptic therapy (VCMs). The cellular oxidative stress hypothesis is another potential physiological and pathological paradigm for TD. Antipsychotic drugs generate neurotoxic free radicals, which are linked to TD. The etiology of TD is thought to include peroxidation. In mice, haloperidol at a dosage of 0.2 mg/kg/day for just five weeks establishes VCMs, which become worse over time as the therapy prolongs. The deterioration in motor control begins after the first week, peaks after three weeks, and then progressively improves to its pre-injury level. A daily intake of 0.4 mL of RBO, a scavenger, reduces the production of haloperidol-induced VCMs and causes motor control to deteriorate, exhibiting the protective barrier of RBO’s antioxidants in the reduction of extrapyramidal signs brought on by haloperidol [149]. Tocotrienols have several important roles, including neuroprotective effects, such as significantly reduced cell damage in cells exposed to hydrogen peroxide in a dose-dependent and time-dependent way. In samples treated with tocopherols, the proportion of cell viability on tocotrienol-treated specimens is considerably more significant. Therapy with tocotrienols strongly reduces axons and neurite degradation, and tocotrienols have a better protection proportion than tocopherols. While tocopherol therapy also has a neuroprotective role due to its antioxidant properties, tocotrienol treatment results in much fewer dendritic and neurite modification regions, including such particle production, contraction, and disintegration, than tocopherol therapy [150].

Because of various preventive benefits in several animal models of neurological conditions, the anti-inflammatory actions of peroxisome proliferator-activated receptors (PPAR) have drawn substantial interest. The polyunsaturated fatty acids, linked to PPAR-γ partially agonist activity, are abundant in RB. RB extract treatment counteracts Lipopolysaccharide’s inflammatory actions by lowering inflammatory mediators’ levels in the brains of mice feeding on RB extract (100 mg/kg) and PG (30 mg/kg). Despite substantially impacting I-κB production or NF-κB binding to its response element, it also lowered PPAR-γ sumoylation. Most benefits diminish when a PPAR-γ antagonist is present, emphasizing that the RBE (agonistic) component’s action on PPAR-γ provides credence to the idea that PPAR-γ activation contributes to the neuroprotective benefit of the RBE [151].

#### 6.5.12. Insomnia Alleviation

Rice bran oil has the potential against insomnia, and it may alleviate its prevalence, as seen through the manipulation of monoamine neurotransmitters. Yang et al. conducted an animal investigation to investigate the impact of high RBO in oryzanol on a pentobarbital-inducing sleep schedule in partially sleep-deprived rats. Sixty rats were randomly split into five divisions (twelve for each class). The controls and PSD models set received basic mice chow with 8 percent soybean oil like a meal (free of oryzanol). The basic rat chow containing 8 percent RBO, comprising 3000, 7000, as well as 15,000 mg per kg of oryzanol, correspondingly, was provided to the low-PSD oryzanol, medium-PSD oryzanol, and high-PSD oryzanol groups over 25 days. The outcome showed that RBO high in oryzanol might lessen tiredness and enhance sleeping in PSD-affected mice via modulating monoamines [152]. An overview of health-endorsing perspectives of RBO is given in Figure 6.

## 7. Industrial Applications

### 7.1. Stabilization of Fats, Frying Oils and Fried Products

The antioxidant and free radical scavenging activities of γ-oryzanol are primarily responsible for RBO’s stabilizing impacts on lipids and oils, which are significant in producing more stable commercial goods as well as the historical usage of RBO as a cooking and salad oil in several Asian nations [149]. Regarding deep frying, weaker, stable liquid lubricants are often hydrolyzed to increase their antioxidant capacity. Moreover, hydroxylation produces large quantities of unwanted trans nutrients plus the positioning of isomer fatty acids. Combining polyunsaturated oil rich in natural scavengers, such as virgin olive and sesame seed oils plus RBO, is a substitute for hydrogenation, significantly improving the durability of foods cooked in all these oils [102]. In rice seed oils generated at various roasted degrees and periods, as roasting temperatures and duration increased, so too the color, phosphorus contents, a-tocopherol, and c-tocopherol quantities increased, yet not the fatty acid and oryzanol amounts [55].

### 7.2. Stabilization and Development of Other Food Products

In addition to being appealing contenders for the development of nutritious foods, brown rice, RB, plus regular and enhanced RBO are utilized to stabilize a range of food items, and by introducing 0.1% RBO, the degradation of reduced heat in whole powdered milk decreased while maintaining production. Buyers could not distinguish a significant flavor difference between the reconstitution of whole powdered milk having 0.1% RBO and standard powdered milk. Additionally, compared to beef patties having various antioxidant properties, γ-oryzanol-carrying beef patties demonstrate improved chemical stability throughout preservation. The beef prepared with γ-oryzanol shows minuscule amounts of C7-oxidized cholesterol, hydrogen peroxide (H2O2), and hexanal, with the least TBARS readings and heated-over taste ratings [153].

### 7.3. Poly Hydroxy Alkenoates

Poly hydroxy alkenoates (PHA) are eco-friendly substitutes for plastic made from petrochemicals that exhibit oxygen barrier qualities on par with polyvinyl chloride and polyethylene terephthalate. Elongated RB with extruded maize starches in varied ratios as the source of carbon and energy for producing PHA can be employed. Different PHA percentages can be made by altering the growing conditions [139]. Uses for PHA include replacing polyethylene and aluminum sheets in producing paper, cardboard, and meal trays. Other key study topics include stitches, wound bandages, bone grafts, general medical disposable items, and medicinal gadgets [131]. Therefore, employing RB to produce PHA can save numerous funds and produce biologically degradable and non-toxic products.

### 7.4. Biodiesels

Because it is harmless, biodegradable, and recyclable, biodiesel has gained more attention. The cost of pure biodiesel is the primary source of worry. The choice of cheap feedstock plus considerable value outputs is among the long-term goals of biodiesel development. RBO is a comparatively cheap raw ingredient used to make biodiesel. The extraction and purity of real-world nutraceuticals produced by biodiesel synthesis from RBO, as well as the use of leftovers like defatted RB to manufacture proteins, carbs, and phytochemicals, are all appealing solutions to reduce the price of biofuels. RBO can be converted into biodiesel by in situ esterification, lipase-catalyzed, acid, and base-catalyzed processes, or other methods [154]. Many derivatives, including oryzanol, RB wax, RB meal, sterols and fatty acids, and lecithin, can indeed be obtained even during the preparation of RBO. Oryzanol, a distinctive product produced from RBO soap stocks, the supply of which essentially only comes from industry, is one of these residues [155].

### 7.5. Miscellaneous Industrial Applications

Rice bran oil is a very cheap raw material used to make biodiesel, and it has the benefit of having the ability to create outputs with a significant added value. As a result, relevant studies have been conducted on creating enzyme-mediated mechanisms. RBO is also used to produce less sticky and more durable biodiesel. Investigations have been conducted on recovering γ-oryzanol, tocols, and other phytonutrients from the leftovers [156]. Red sea bream, feeding on commercially brown fish meals enriched between either trans-ferulic acids (0.01–0.5%) or γ-oryzanol, shows a more vibrant minimal measure color. Additionally, such fish possess lower levels of TBARS in their livers than reference fish, showing that in cultivated red sea breams, trans-ferulic acids plus c-oryzanol inhibit oxidative damage in addition to dark-color pigmentation [157]. Ortho, meta, and para dichlorobenzenes have been used as pesticides for decades. Para-isoform is an example of being employed extensively to control pests and moths that infest clothing, skins, furs, and museum items. Surprisingly, RB is a robust para-dichlorobenzene absorber throughout an extensive pH range of 1 to 12. A Freundlich-type desorption effect is observed, and a characteristic of RB is related to the absorption by spherosomes and cellular granules [157].

## 8. Conclusions

Rice bran is an underutilized by-product of rice milling—extracting and refining RBO captures some inherent value. RBO is considered one of the most refined vegetable oils available, with an anticipated production of 722.2 metric tons. Japan, China, and India are widely recognized as the leading nations in manufacturing RBO. Small-scale rice milling processes over 50% of the total rice production. Therefore, there is still a potential availability of 20–25 million metric tons of bran for oil extraction. RBO has been found to possess cholesterol-lowering properties compared to other highly unsaturated oils due to its rich content and diverse antioxidants. The taste and practicality of this product complement its suitability for inclusion in salads, culinary preparations, and frying applications. Refined RBO offers a range of health and nutritional advantages, including an anti-hyperlipidemic effect, anti-diabetic effect, anti-cancer effect, anti-hypertensive effect, and anti-aging effect, and cholesterol-lowering effects, neuroprotective effects, immunoregulatory effects, effects on colorectal cancer, effects on muscles, effects on menopause, cosmetic uses, and various industrial applications. It is advisable to use RBO in our everyday cooking practices to obtain optimal health.

Regarding limitations, there could be purity issues while extracting rice bran oil from the bran. Solvent residues could be present in the final product, but these issues could be limited if the process is completed carefully.

## Figures and Tables

**Table 1:** Various stabilizing methods with their advantages and disadvantages.

Methods	Mode of Action	Disadvantages	Advantages	References
Microwave heating	Heat energy from the microwave is converted to denature the lipase enzymes.	Expensive and unsuitable for remote regions. Decreased fiber level.	Simple penetration, simultaneous heating, quick heating, and even heating. Improved bioavailability of vital compounds.	[17,18]
Extrusion	High temperature, pressure, and shear force all contribute to inactivation.	Because strong extrusion conditions might damage nutrients, operational conditions must be strictly controlled.	High output, quick processing time, effective antioxidants, and essential nutrients are maintained throughout inactivation.	[19]
Dry heating	Temperature range: 100–130 °C. Hot air reduces moisture content, resulting in lipase deactivation.	Water reabsorption has the potential to lessen the inhibitory impact. Only suitable for small-scale applications	Simple method. The nutritional potentials of the extracts were preserved.	[20]
Infrared heating	Radiation enters the substance and is transformed into heat.	Radiation penetration is limited, resulting in less uniform heating. Radiation may have an impact on the stability of vital nutritional components.	Versatile and quick reaction Lipase deactivation is efficacious.	[20]
Low-temperature treatment	Bran preservation at a low temperature (0 °C) regulates lipase activity.	Incomplete suppression owing to activity resumption at room temperature Only suitable for small-scale use. Maintaining a low temperature is wasteful in terms of cost.	Does not influence nutritional availability.	[21]
Ohmic heating	An alternating electric current was transmitted through the rice bran, which acted as electrical resistance, creating heat and inactivating the lipase enzyme.	An electric field can influence the metabolic processes.	Heating that is consistent Lipase enzyme inactivation is effective. Increased accessibility to essential molecules like γ-oryzanol and tocopherols.	[22]
Biological treatment	Break down the hydrolytic enzyme lipase with another enzyme, for example, protease.	The enzymes are expensive to obtain.	Allow for the specific targeting of enzyme activity. Essential nutritional components are preserved.	[23]
Moisture heating	Hot steam inactivates lipase.	Polyphenol concentration has been reduced.	Inactivation efficiency, extended storage period, consistent heating, quick heating, nutritious components preserved	[24]

**Table 2:** Comparison of conventional and non-conventional methods.

Techniques	Parameter	Optimized Condition	Finding and Yield	References
**Ultrasound-assisted extraction (UAE)**	Time Solvent used Frequency Temperature Solid to liquid ratio	60 min Petroleum ether, Hexane and Methanol 24 kHz 38 °C 1:3	Compared to the usual extraction approach, ultrasound treatment considerably improves methanol’s γ-oryzanol extraction performance. Extraction efficiency was 96% of the total oil content available in rice bran.	[35]
**Microwave-assisted extraction (MAE)**	Time Power Solvent used Solid to liquid ratio	30, 60, 90 and 120 s per step 300, 500, 700, and 900 W Hexane 1:1	MAE is a potential method for extracting RBO with higher oil purity, increased oil production, and reduced extraction duration. Extraction efficiency with microwave is 80% of the total oil content available in rice bran.	[36]
**Soxhlet extraction**	Temperature Solvent	65 °C Hexane	The extraction of solvents Hexane as a solvent is an efficient approach for RBO extraction. The extraction efficiency is 80–90% of the total oil content available in rice bran, while the solvent is n-hexane.	[37]
**Supercritical CO_2_ extraction**	Time Pressure Temperature Solvent used Solid to liquid ratio	30 min 100, 150, or 200 bar 40 °C, 60 °C or 80 °C Ethanol 0:1, 0.5:1, 1:1, 2:1	The goal of supercritical CO_2_ may be to reduce operational expenses while increasing oil output. The extraction efficiency of rice bran oil is 25–26% by weight basis.	[38]
**Enzyme-assisted three-phase partitioning**	Three phases	t-butanol (top phase), protein (middle phase), and ammonium sulfate (lower phase)	This technique was initially used to separate proteins, enzymes, and lipids but is now used to extract bioactive such as oils, oleoresins, and polysaccharides from plant sources. Rice bran extraction efficiency is 79% of total oil content using Proteases and Protizyme.	[39]
**Sub-critical water extraction**	Time of ultrasound Temperature Solvent used Solid to liquid ratio	10–20 min 180–240 °C Deionized water 1:6	Subcritical water extraction is a low-impact approach for lipase deactivation and RBO stabilization. The extraction efficiency of rice bran oil is 249 mg/g on a dry matter basis at 240 °C in pure water.	[40]
**Solvent extraction**	Temperature Time Solvent used Solid to liquid Ratio	60 °C, 40 °C 10 min Hexane, Isopropanol 2:1, 3:1	The solvent extraction process in the industry can achieve a high yield and recovery rate of as much as 99%.	[19]

**Table 3:** Therapeutic profile of rice bran oil.

Micronutrient	Amount %	Advantage
Oryzanol	1.2–1.7	Increase good (HDL) cholesterol and decrease. bad (LDL) cholesterol, treats nerve imbalance and menopause disorder, anti-aging effects, antidandruff and anti-itching agent
Tocotrienol	0.025–0.17	Cholesterol reduction, reversing. atherosclerosis, anti-cancer (breast, liver) tumor suppression, antioxidant
Tocopherol	0.02–0.08	Antioxidant, free radical scavenger, reduce risk of cardiovascular diseases, arthritis, cancer and cataracts, anti-tumor activities.
Squalene	0.3–0.4	Antioxidant

**Table 4:** Bioactive compounds from rice bran oil and their biological potentials.

Secondary Metabolite	Biological Potentials	References
Trans-ferulic acid	Antioxidant	[91]
Cis-ferulic acid	Antioxidant	[91]
Vanillic aldehyde	Antioxidant	[91]
Caffeic acid	Antioxidant	[92]
Chlorogenic acid	Antioxidant	[92]
Gallic acid	Antioxidant	[92]
Syringic acid	Antioxidant	[92]
Tricin	DPPH radical scavenging activity	
Gramisterol	Anti-cancer activity	[93]
Lupeol	Anti-cancer activity	[93]
24-methylene cycloartenol	Lowering postprandial hyperglycemia.	
24-methyl cholesterol cis-ferulate	Anti-inflammatory activity	[94]
Stigmastanol cis-ferulate	Anti-inflammatory activity	[95]
Cycloeucalenol	Anti-cancer activity	[93]

**Table 5:** Health indorsing perspective of rice bran oil.

Amount Ingested of RBO	Model	Results	References
400 mg/kg-Tocotrienols	Mice	Gamma-tocotrienol of RBO has the potential to reduce pancreatic. Tumor growth by inhibiting the NF-KB-mediated inflammatory. microenvironment	[101]
30 mL	A randomized double-blind control trial	Gamma-oryzanol-rich RBO may improve cardiovascular. disease risk factors by decreasing LDL-C levels and increasing antioxidant potential in hyperlipidemic issues	[102]
2 mL/kg	Hypertensive rats	γ-oryzanol-rich RBO provides a protective mechanism against oxidative stress and hypertension	
50 g/100 g	Male mice	RBO regulates inflammatory responses in murine macrophages. by upregulating mitochondrial respiration	[103]
100 mg/kg	Male Kunming mice	γ-oryzanol protects against ethanol-induced liver injury, which might be due to its alleviation of oxidative stress and inhibition of apoptosis, possibly inhibiting MAPK. Signaling pathways mediated mitochondrial signaling pathway activation. Rice bran contains immune system-boosting components. including phytosterols, sterol ins and gamma-oryzanol, omega-3 acids, phytonutrients, minerals, etc.	[104]
100 mg kg^−1^ day^−1^	Mice	γ-oryzanol, a component of RBO, shows an anti-allergic effect to inhibit the allergy by reducing the action of NF-KB	

## Data Availability

No new data were created or analyzed in this study. Data sharing is not applicable to this article.
